# Global diversity and distribution of nitrogen-fixing bacteria in the soil

**DOI:** 10.3389/fpls.2023.1100235

**Published:** 2023-01-20

**Authors:** Siim-Kaarel Sepp, Martti Vasar, John Davison, Jane Oja, Sten Anslan, Saleh Al-Quraishy, Mohammad Bahram, C. Guillermo Bueno, Juan José Cantero, Ezequiel Chimbioputo Fabiano, Guillaume Decocq, Rein Drenkhan, Lauchlan Fraser, Roberto Garibay Oriel, Inga Hiiesalu, Kadri Koorem, Urmas Kõljalg, Mari Moora, Ladislav Mucina, Maarja Öpik, Sergei Põlme, Meelis Pärtel, Cherdchai Phosri, Marina Semchenko, Tanel Vahter, Aida M. Vasco Palacios, Leho Tedersoo, Martin Zobel

**Affiliations:** ^1^ Institute of Ecology and Earth Sciences, University of Tartu, Taru, Estonia; ^2^ Zoology Department, College of Science, King Saud University, Riyadh, Saudi Arabia; ^3^ Department of Ecology, Swedish University of Agricultural Sciences, Uppsala, Sweden; ^4^ Universidad Nacional de Córdoba, Instituto Multidisciplinario de Biología Vegetal, Consejo Nacional de Investigaciones Científicas y Técnicas (CONICET), Córdoba, Argentina; ^5^ Universidad Nacional de Río Cuarto, Departamento de Biología Agrícola, Facultad de Agronomía y Veterinaria, Córdoba, Argentina; ^6^ Department of Wildlife Management and Ecotourism, University of Namibia, Katima Mulilo, Namibia; ^7^ Ecologie et Dynamique des Systèmes Anthropisés (EDYSAN, UMR CNRS 7058), Jules Verne University of Picardie, Amiens, France; ^8^ Institute of Forestry and Engineering, Estonian University of Life Sciences, Tartu, Estonia; ^9^ Department of Natural Resource Sciences, Thompson Rivers University, Kamloops, BC, Canada; ^10^ Instituto de Biología, Universidad Nacional Autónoma de México, Ciudad de México, Mexico; ^11^ Iluka Chair in Vegetation Science and Biogeography, Harry Butler Institute, Murdoch University, Perth, Australia; ^12^ Department of Geography & Environmental Studies, Stellenbosch University, Stellenbosch, South Africa; ^13^ Center of Mycology and Microbiology, University of Tartu, Tartu, Estonia; ^14^ Department of Biology, Nakhon Phanom University, Nakhon Phanom, Thailand; ^15^ Grupo de Microbiología Ambiental y Grupo BioMicro, Escuela de Microbiología, Universidad de Antioquia UdeA, Medellín, Colombia; ^16^ Department of Botany, Institute of Ecology and Earth Sciences, University of Tartu, Tartu, Estonia

**Keywords:** 16S SSU, bacterial diversity, biological N fixation, N-fixing bacterial community, biotic interactions, nifH gene

## Abstract

Our knowledge of microbial biogeography has advanced in recent years, yet we lack knowledge of the global diversity of some important functional groups. Here, we used environmental DNA from 327 globally collected soil samples to investigate the biodiversity patterns of nitrogen-fixing bacteria by focusing on the *nif*H gene but also amplifying the general prokaryotic 16S SSU region. Globally, N-fixing prokaryotic communities are driven mainly by climatic conditions, with most groups being positively correlated with stable hot or seasonally humid climates. Among soil parameters, pH, but also soil N content were most often shown to correlate with the diversity of N-fixer groups. However, specific groups of N-fixing prokaryotes show contrasting responses to the same variables, notably in Cyanobacteria that were negatively correlated with stable hot climates, and showed a U-shaped correlation with soil pH, contrary to other N-fixers. Also, the non-N-fixing prokaryotic community composition was differentially correlated with the diversity and abundance of N-fixer groups, showing the often-neglected impact of biotic interactions among bacteria.

## Introduction

Nitrogen limitation to net primary production is widespread in terrestrial and marine ecosystems (Vitousek & Howarth, 1991), and biological nitrogen fixation has a major role in providing nitrogen to the ecosystem. In biological fixation, gaseous N2 is assimilated and transformed only by a select group of microorganisms, either plant symbionts or free-living diazotrophs (Pajares & Bohannan, 2016). These microorganisms are capable of expressing the nitrogenase enzyme codified by *nif* genes. One of these, the *nif*H gene coding nitrogenase reductase, has been accessed with molecular techniques for studies on microbial communities’ potential to fix atmospheric N2 (Gaby & Buckley, 2014). The particular microbes include i) bacteria collectively referred to as rhizobia (e.g., Rhizobiaceae, Burkholderiaceae), ii) Actinobacteria from the genus Frankia, iii) Cyanobacteria from the Nostocaceae family, but also iv) free-living Bacteria and Archaea that have obtained nitrogenase *via* horizontal transfer (Kuyper & de Goede, 2013; Vitousek et al., 2013). Rhizobiaceae (α-proteobacteria) and Burkholderiaceae (β-proteobacteria) are the most well-known N-fixing bacterial groups that nodulate mostly on legumes, although a few other plant genera can host rhizobia as well (Peix et al., 2015; Sprent et al., 2017; Tedersoo et al., 2018). Nitrogen-fixing Frankiaceae inhabit root nodules of eight angiosperm families, and the mutualistic Frankia species fall into three genetic lineages (Normand et al., 2007). In addition, Frankia are considered to be ubiquitous free-living soil organisms because many taxa have been recorded outside the distribution range of their compatible hosts (Chaia et al., 2010). Several groups of Cyanobacteria also possess the ability to fix N2. Although well suited to independent existence in nature, some Cyanobacteria occur in symbiosis with a wide range of hosts, including protists, animals, and plants (Rai et al., 2000).

The *nif*H gene is the biomarker most widely used to study the ecology and evolution of nitrogen-fixing bacteria (Gaby & Buckley, 2014). Surveys of *nif*H diversity conducted in a wide range of environments (Izquierdo & Nüsslein, 2006; Põlme et al., 2014; Penton et al., 2016; Wang et al., 2017; Nash et al., 2018; Silveira et al., 2021; Zhou et al., 2021) have demonstrated that the diversity and community composition of N-fixing bacteria vary significantly across habitats and regions. However, we lack a systematic understanding of the global distribution and diversity patterns of these bacteria and the environmental factors underlying them.

According to Tedersoo et al. (2018) and Tamme et al. (2021), climatic factors significantly drive the diversity of the N-fixing host plants. They found that the absolute diversity of N-fixing plants is highest in warm and wet climates, while the relative richness of N-fixing plants (share in the local flora) is highest in warm and dry climates. We expect the same is true for symbiotic rhizobia. However, symbiotic Actinobacteria and Cyanobacteria, as well as the total N-fixing prokaryotic community, may exhibit different patterns. Plants in symbiosis with Actinobacteria are more abundant at high latitudes (Menge et al., 2017; Tamme et al., 2021), while the host plants for N-fixing Cyanobacteria occur both in the tropics as well as in the boreal zone (DeLuca et al., 2002; Sprent et al., 2017). Little is known about the global distribution of free-living N fixers, however, although for example in boreal ecosystems, tropical rain forests, temperate grasslands and arctic tundra, free-living nitrogen-fixation might outweigh symbiotic N-fixation (Reed et al., 2011).

The diversity of N-fixing plants varies across biomes: Tedersoo et al. (2018) and Tamme et al. (2021) found that the relative richness of N-fixing plants is highest in tropical and temperate grasslands and semi-deserts. The same pattern may also hold concerning N-fixing bacteria. In addition, the distribution of host plants exhibits regional variation. For instance, the absolute diversity of N-fixing plants is very high in Australia (Tamme et al., 2021). Moreover, Sprent et al. (2017) provide a thorough overview of the biogeographic history and demonstrate that regionally unique evolutionary histories of particular clades of bacteria certainly contribute to the regional turnover of the composition of mutualistic bacterial communities. Part of the regional effect on microbial diversity may therefore be specifically related to the historical distribution of biomes (Pärtel et al., 2017), but there are no data with which to assess its effect on N-fixing organisms. In addition, recent studies have shown that biotic interactions between soil microbes may significantly structure their communities (Bahram et al., 2018; Soliveres et al., 2018). However, we are unaware of any work studying the impacts of host plants, historical biome distribution or soil biotic interactions on N-fixing bacteria.

Given the significant effect of soil pH on bacterial communities in general, notably the increase in the abundance and diversity of bacteria with rising soil pH (Lauber et al., 2009; Bahram et al., 2018; Delgado-Baquerizo et al., 2018), we hypothesised that soil pH also affects the N-fixing bacteria positively. We also expected variation in N-fixing bacterial community composition to associate with nitrogen availability. In experiments, the abundance of nitrogen-fixing bacteria tends to be suppressed by fertilisation with N and increased by fertilisation with P, while both alter the taxon composition of the bacterial communities (Wang et al., 2017). Analogous responses might be expected along natural fertility gradients.

Here, we use environmental DNA (eDNA) extracted from 327 spatially-distinct soil samples from all continents except Antarctica to provide a first overview of the pattern of global biodiversity of N-fixing bacteria. We primarily addressed the distribution of N-fixing bacteria by analysing the *nif*H gene, but the bacterial 16S SSU rRNA (small subunit ribosomal RNA) gene was also used for complementary analyses.

## Materials and methods

### Sampling and data collection

We used a global set of 327 soil samples (**Figure S1**; **Table S1**) compiled from two similar global sampling efforts (dataset described in Davison et al., 2020). Briefly, sampling locations generally experienced little disturbance from human activities, from where topsoil (1-5 cm, to enable a uniform sampling depth considering locations with very shallow soils) samples were collected after removing the litter layer. The sampling was conducted following one of the two approaches: a) about 300 g of topsoil was collected from up to 40 points within about a 50 × 50 m sampling area and then pooled [233 samples]; or b) per sampling area, 5 g topsoil samples were collected from nine points on a regularly spaced 30 × 30 m grid and then pooled [94 samples]. The sampling design was included in statistical models as a covariate. Soil samples were dried within 24 h using silica gel at room temperature. Subsamples [a) 2 g; b) 5 g] of soil were extracted for molecular analysis; the remainder was stored for chemical analysis.

Soil chemical properties were measured from sieved soil samples (2 mm): pH, total N, P, K, Mg, and Ca. Soil pH was measured in 1 M KCl solution following ISO 10390:2005, using a Seven Easy pH meter with an InLab Expert Pro electrode (Mettler Toledo, Malaysia). The content of total N in soil was determined using the Kjeldahl method with a DK-20 digestion block and a UDK-126 distillation unit (Velp Scientifica Srl, Italy). For the determination of P, K, Mg, and Ca, the Mehlich III extraction method was used, with the content of elements determined using an MP-4200 microwave plasma atomic emission spectrometer (Agilent, USA). Chemical analyses were performed at the Institute of Agricultural and Environmental Sciences, Estonian University of Life Sciences, Tartu, Estonia. In further data analyses, and due to collinearity issues, K, Mg and Ca were ln-transformed and standardised (mean = 0, SD = 1). Then we performed a principal component analysis (PCA) with these macronutrients. In subsequent analyses, we incorporated the first principal component, which described 84.6% of the combined variance, and is negatively correlated to the amount of K, Mg and Ca in soil, referred to as “Other soil macronutrients” henceforth. The rest of soil chemical properties, i.e. total N, P, K, were included and analysed as individual factors.

Bioclimatic variables for each sampling location were accessed from the CHELSA high resolution modelled world climate database (Karger et al., 2017). To incorporate as much of the background climatic information without overparameterizing the model, we performed PCA on the climate variables, standardised before the analysis (precipitation variables were *ln*-transformed), and included the first three principal components in subsequent analysis. The first principal component (Bioclim PC1) correlated with a stable hot climate, the second (Bioclim PC2) with a warm, arid climate, and the third (Bioclim PC3) with a seasonal humid climate (**Figure S2**). The first three principal components described approximately 79% of the total variation in bioclimatic factors.

Sampling locations were assigned to biogeographic realms following Olson et al. (2001). We combined ecologically similar ‘biomes’, resulting in seven biome combinations (following Tamme et al., 2021; **Table S2**). The historical stability of biomes at sampling locations was estimated by comparing the Olson et al. (2001) current biome classification with an analogous classification for the Last Glacial Maximum (LGM; approximately 21kyBP; Ray & Adams, 2001). Where the biome remained the same at both times, the sampling location was classified as stable; where the biome classification differed, the sampling location was classified as unstable.

The richness of N-fixing plants was estimated using data from Tamme et al. (2021). In short, GBIF records (GBIF.org, 2017) falling within roughly 7800 km² hexagonal grid cells surrounding the sampling coordinates were checked for quality and identified as N-fixing and non-fixing plant species based on the NodDB database (Tedersoo et al., 2018), and used for richness calculations.

### Molecular methods and bioinformatics

DNA was extracted from either a) 2 g or b) 5 g of dried soil using the PowerMax^®^ Soil DNA Isolation Kit (MoBio Laboratories, Carlsbad, California, USA). Two complementary DNA regions were used to identify N-fixing microorganisms, the dinitrogenase reductase subunit *nifH* gene and the general prokaryotic 16S rRNA gene.

#### *nif*H gene

The specific primer pair 19F (5′-GCIWTYTAYGGIAARGGIGG-3′) and 407R (5′-AAICCRCCRCAIACIACRTC-3′) (Ueda et al., 1995) was used for amplifying the *nif*H gene. Primers were equipped with Illumina Nextera XT sequencing tags. PCR was carried out in the following thermocycling conditions: an initial 15 min at 95°C, followed by 38 cycles of 95°C for 30 s, 55°C for 30 s, 72°C for 1 min, and a final cycle of 10 min at 72°C. The amplicons were ligated using Illumina Nextera XT sample preparation kit (Illumina Inc., San Diego, USA) following the manufacturer’s protocol. Ligation of Illumina adaptors and sequencing using Illumina MiSeq 2x250 bp paired-end mode were performed at the Estonian Genome Centre (Tartu, Estonia).

The data were analysed using the gDat pipeline (Vasar et al., 2021). Demultiplexed paired-end reads were analysed in the following way: primer sequences were matched, allowing 1 mismatch for both pairs. Only pairs where both reads had an average quality score of >30 were retained (after removal of primer sequence). Quality filtered paired-end reads were combined using FLASh (v1.2.10; Magoč & Salzberg, 2011) with the default parameters (10-300 bp overlap with at least 75% identity). Orphan reads (paired-end reads that did not meet the conditions for combination) were removed from the analyses, leaving 7,153,685 cleaned combined sequences. The VSEARCH (v2.21.1; Rognes et al., 2016) chimera filtering algorithm was used to remove putative chimeric reads in the reference mode using the *nif*H database (Gaby & Buckley, 2014), yielding 5,083,833 chimera-free sequences that were clustered at 99% identity (following the recommendation of Edgar, 2018) into 65,713 OTUs (excluding OTUs with less than 10 sequences). Representative sequences (OTU centroids) were taxonomically classified using a BLAST+ search with ≥90% identity threshold against the *nif*H database using the best hit, resulting in 14,957 OTU hits (996,027 sequences) which were used in downstream analysis as the total N-fixer community. To enable taxonomic identification of specific groups of N-fixing organisms, we run an additional BLAST+ search against a subset of the *nif*H database sequences with at least family-level identity known using the best hit, resulting in 9332 OTU hits (565,921 sequences).

#### 16S rRNA

Prokaryotic primers 515F (5′-GTGYCAGCMGCCGCGGTAA-3′) and 926R (5′-GGCCGYCAATTYMTTTRAGTTT-3′) were used to amplify the 16S rRNA variable V4 region (Caporaso et al., 2011; Parada et al., 2016). Both primers were equipped with unique 12-base Golay barcodes for multiplexing. The PCR amplification program included the following steps: 95°C for 15 min, followed by 25 cycles of 95°C for 30 s, 55°C 30 s and 72°C for 1 min, with a final extension step at 72°C for 10 min. The PCR products were pooled and visualised on 1% agarose gel. Initially, 25 cycles were used for all the samples, and in case the gel band was weak, or there was no PCR product, a higher number of PCR cycles was used (max 30 cycles). Negative and positive controls were included throughout the process. The PCR products were purified using a FavorPrepTM GEL/PCR Purification Kit (Favorgen Biotech Corporation). The libraries were ligated with Illumina adaptors using the TruSeq DNA PCR-free library prep kit (Illumina Inc., San Diego, CA, USA). Libraries were sequenced on the Illumina MiSeq platform, using a 2 × 250 bp paired‐read sequencing approach at Asper Biogene (Tartu, Estonia).

The data were analysed using the gDAT pipeline (Vasar et al., 2021) with the same parameters and thresholds used for the *nif*H gene analysis. Orphan reads (paired-end reads that did not meet the conditions for combination) were removed from the analyses, leaving 14,593,006 cleaned combined sequences. The VSEARCH chimera filtering algorithm was used to remove putative chimeric reads in *de novo* mode, yielding 13,960,691 chimera-free sequences clustered at 99% identity into 165,330 OTUs (excluding singletons). Representative sequences (OTU centroids) for each non-singleton OTU were taxonomically classified using a BLAST+ search against the SILVA database (v132; Quast et al., 2013), taking the lowest common ancestor using the top 50 best hits (i.e., using the most precise taxonomic resolution at which the top 50 best hits converge), resulting in 141,959 hits.

#### N-fixer groups

Subsets of N-fixing organisms were defined according to their taxonomy: rhizobia (genus field containing “*rhizobium|Rhizobium”), Cyanobacteria (phylum field containing “Cyanobacteria”) and Frankia (genus field containing “Frankia”). The paraphyletic rhizobia were limited only to the mentioned extent due to the limited availability of species-level taxonomic identification of the OTU-s, precluding the incorporation of nodule-forming species from genera with several different trophic modes (Willems, 2006). For the *nif*H dataset, we also used the total N-fixers, incorporating all the OTU-s from that primer set that had passed all quality filters and BLAST+ identity thresholds against the full *nif*H database. OTU-s from the 16S SSU dataset not included in the three N-fixer groups were used in further analyses to represent the non-N-fixing prokaryotic community, referred to as “prokaryotic community composition” henceforth.

Raw reads from this Targeted Locus Study have been deposited in the National Center for Biotechnology Information Sequence Read Archive (NCBI SRA; BioProject PRJNA659159). The environmental metadata and community data matrices have been deposited in Mendeley Data (doi: 10.17632/dsvcw24cyc.1).

### Statistical analysis

We tested the effects of climate, soil chemical parameters, richness of N-fixing plant species, biome and historical biome stability, and composition of other prokaryotes on the natural logarithm of richness, Shannon diversity index, and relative abundance [ln(abundance of N-fixer group/(abundance of all prokaryotes including the N-fixer group – group abundance))] of different groups of N-fixing bacteria by fitting linear models using Generalised Least Squares (gls() from R package nlme v3.1-157; Pinheiro et al., 2021). Model predictors were tested for collinearity using Variance Inflation Factors (vif() from R package car v3.0-13; Fox & Weisberg, 2019), following which the biome variable was omitted from further analyses. The sampling design, i.e., from which of the two sampling campaigns the sample originated, was also included in the models as a covariate. To account for potential differences arising from unequal sampling depths (i.e., number of sequences obtained from a sample), we used Hill numbers of order 0 and 1 (richness and Shannon diversity [exp(*H*)], respectively) that were extrapolated to the asymptote to estimate the diversity at complete sampling coverage, using the R package iNEXT v2.0.20 (Hsieh et al., 2016). After extrapolation, the Shannon diversity [exp(*H*)] was log-transformed similar to richness, yielding the Shannon diversity index (*H*). For readability, we refer to the extrapolated diversity metrics as “richness” and “Shannon diversity” throughout the paper. The models incorporated a spherical spatial correlation structure with great circle distances. The models were run in parallel with data from both genes, *nif*H and 16S SSU, except for the N-fixer group relative abundances, which could be calculated only for the 16S SSU dataset. Soil pH, P, N, soil chemistry principal component 1, and bioclimatic principal components were included in the model as linear and quadratic terms to capture unimodal relationships. Drivers of N-fixer communities were identified using distance-based redundancy analysis (dbRDA() from R package vegan v2.6-2; Oksanen et al., 2020) on Hellinger-transformed OTU abundances.

To visualise the biogeographical patterns in the richness of N-fixer groups, we generated interpolated maps using weighted categorical k-nearest neighbour (KNN) classification (kknn() in the R package kknn v1.3.1; Schliep & Hechenbichler, 2016), using the soil sample richnesses as the training set and a 0.5° × 0.5° map grid as the test set. Grid cell value represents the weighted value of the k nearest training set cells based on great-circle distances. The k-value (k = 18) for KNN was set as the rounded square root of the number of samples, based on the suggestion of (Duda et al., 2000). Greenland and the Sahara region were excluded from the interpolation due to insufficient sampling and differing abiotic conditions.

All statistical analyses were performed in the R language (v4.2.1) in the RStudio IDE (R RStudio Team, 2016; Core Team, 2021).

## Results

In the *nif*H dataset, we identified a total of 14,957 OTUs as N-fixing microorganisms, and in the taxonomically annotated subset, we identified 5810 rhizobia, 619 Cyanobacteria, and 28 Frankia OTU-s. The 16S SSU dataset yielded 1560 rhizobia, 510 Cyanobacteria, and 27 *Frankia* OTUs. Due to the very low yield of Frankia sequences and richness in both datasets (e.g., *nif*H *Frankia* richness: **Figure S3**), *Frankia* were not analysed separately but were only included within the total N-fixers group.

### N-fixer richness

**Figure 1:**
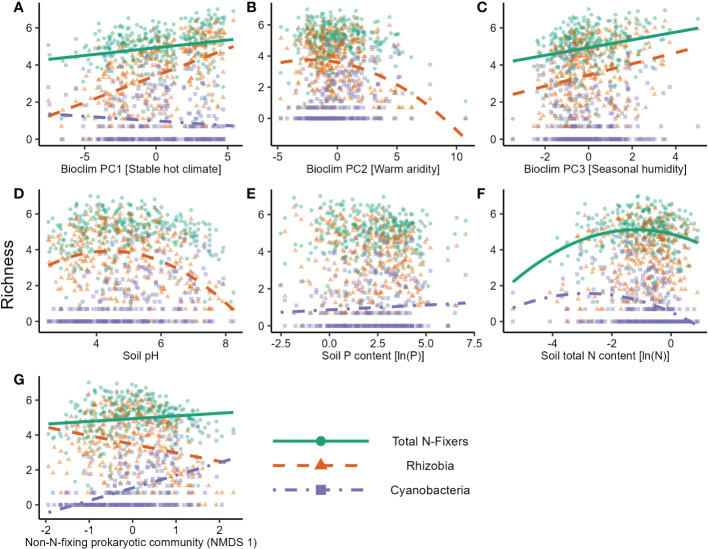
Factors affecting the natural logarithm of richness of different groups of N-fixing prokaryotes in soil samples in the nitrogenase reductase nifH gene dataset. Lines show predicted values from the GLS model. The effects of **(A)** Bioclim PC1 [Stable hot climate], **(B)** Bioclim PC2 [Warm aridity], **(C)** Bioclim PC3 [Seasonal humidity], **(D)** soil pH, **(E)** soil P content, **(F)** soil total N content, and **(G)** the community composition gradient of the non-N-fixing prokaryotic community (NMDS axis 1) are shown.

**Figure 2:**
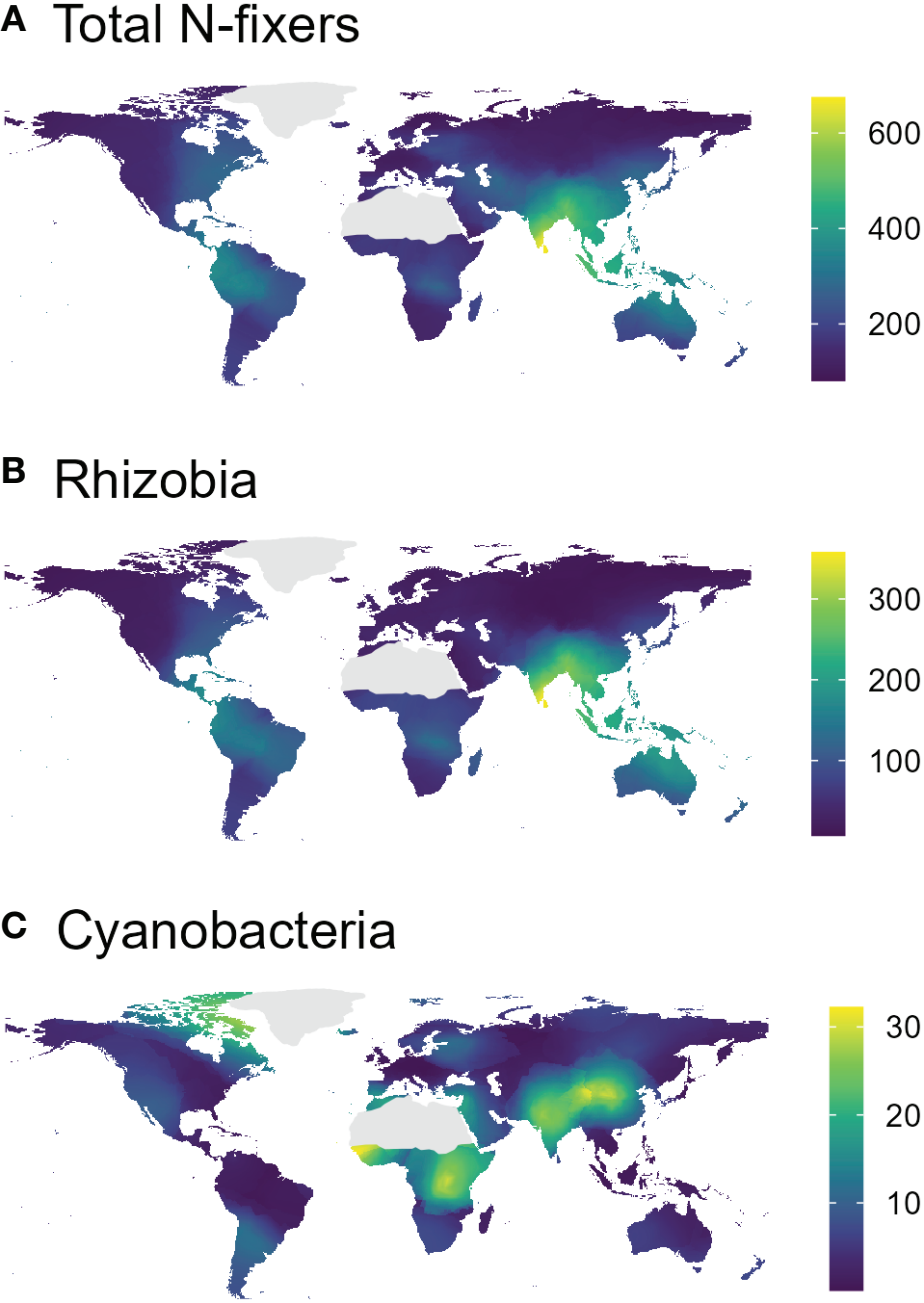
Interpolated (k-nearest-neighbour map cell interpolation based on the values from the collected samples, cell size = 0.5°×0.5°, k = 18) richness maps of total N-fixers **(A)**, rhizobia **(B)**, and Cyanobacteria **(C)**, based on the nifH sequencing dataset.

In the *nif*H dataset, stable hot climates favoured the richness of total N-fixers and rhizobia but were negatively associated with Cyanobacteria richness (**Figure 1A**; **Table 1**), whereas rhizobia richness was unimodally correlated with warm, arid climates (**Figure 1B**; **Table 1**). Seasonal humidity positively correlated with total N-fixer and rhizobia richness (**Figure 1C**; **Table 1**). Total N-fixers and rhizobia richness were also higher at greater latitudes and in locations where the biome has historically reimained stable when compared to the Last Glacial Maximum (**Table 1**). Intermediate soil pH levels favoured rhizobia richness (**Figure 1D**; **Table 1**), and intermediate values of soil total N content favoured total N-fixers and Cyanobacteria (**Figure 1F**; **Table 1**); soil P content was positively associated only with Cyanobacteria richness (**Figure 1E**; **Table 1**). N-fixer richness was also associated with the non-N-fixing prokaryotic community, with total N-fixer and Cyanobacteria richness increasing and rhizobia richness declining along the main axis of variation in prokaryotic community composition (**Figure 1G**; **Table 1**). Similar trends were apparent using N-fixer Shannon diversity (**Table S4**; **Figure S4**) as the response variable, with the exceptions that total N-fixer Shannon diversity was unimodally correlated with soil pH in addition to rhizobia, and rhizobia were unimodally related to soil N content. The 16S SSU dataset also generally exhibited similar patterns (**Table S3**) but revealed a U-shaped response of Cyanobacteria richness and Shannon diversity (**Table S4**) to soil pH that was not apparent in the *nif*H data. The global patterns of N-fixing microorganism richness in the *nif*H dataset are visualised on **Figure 2**.

**Table 1:** Factors affecting the natural logarithm of richness of different groupings of N-fixing prokaryotes in the nitrogenase reductase *nif*H dataset.

	Full *nif*H	Rhizobia	Cyanobacteria
Prokaryotic community composition	4.53 ± 0.21 ***	−0.42 ± 0.19 *	0.67 ± 0.18 ***
Absolute latitude	0.43 ± 0.16 *	0.02 ± 0.008 *	NS
pH	NS	−4.93**²** ± 1.43 ***	NS
ln(P)	NS	NS	4.52 ± 1.64 **
ln(N)	−3.0**^2^** ± 1.12 **	NS	−3.4**^2^** ± 1.27 **
Bioclim PC1 [Stable hot]	8.54 ± 2.28 ***	18.32 ± 2.67 ***	−6.32 ± 2.57 *
Bioclim PC2 [Warm aridity]	NS	−3.38**²** ± 1.38 *	NS
Bioclim PC3 [Seasonal humidity]	4.34 ± 1.18 ***	5.38 ± 1.38 ***	NS
Other soil macroelements [K, Ca, Mg]	NS	NS	NS
N-fixing plant richness	NS	NS	NS
Historical stability of biome	0.49 ± 0.21 *	0.50 ± 0.24 *	NS

Linear model (using Generalised Least Squares) parameter estimates with corresponding standard errors are shown, **²** following the effect size denotes the effect of the quadratic term. P values are reported as follows: NS, not significant; *p < 0.05; **p < 0.01; ***p < 0.001.

### N-fixer relative abundance

Cyanobacterial relative abundance showed a U-shaped response to a warm, arid climate (**Table 2**; **Figure S5A**). The relative proportion of rhizobia exhibited a unimodal relationship with soil pH (**Table 2**; **Figure S5B**) and a negative relationship with soil P content (**Table 2**; **Figure S5C**). The proportion of Cyanobacteria, on the contrary, demonstrated an overall negative but a U-shaped quadratic response to soil pH (**Table 2**; **Figure S5B**) and soil total N content (**Table 2**; **Figure S5D**). The relative abundance of rhizobia and Cyanobacteria showed contrasting relationships with the community composition of non-N-fixing prokaryotes (**Table 2**; **Figure S5E**).

**Table 2:** Factors affecting the relative abundance [ln(group/(all prokaryotes – group))] of rhizobia and Cyanobacteria.

	Rhizobia	Cyanobacteria
Prokaryotic community composition	−0.49 ± 0.10 ***	1.14 ± 0.18 ***
Absolute latitude	NS	NS
pH	−3.66**²** ± 0.78 ***	−11.84 ± 2.18 *** 2.85**^2^** ± 1.20 *
ln(P)	−2.75 ± 0.94 **	NS
ln(N)	NS	−6.58 ± 1.51 *** 2.30**^2^** ± 1.11 *
Bioclim PC1 [Stable hot]	NS	NS
Bioclim PC2 [Warm aridity]	NS	4.36**^2^** ± 1.16 ***
Bioclim PC3 [Seasonal humidity]	NS	NS
Other soil macroelements [K, Ca, Mg]	NS	NS
N-fixing plant richness	NS	NS
Historical stability of biomes	NS	NS

The relative proportions were calculated for the general prokaryotic 16S SSU dataset. Linear model (using Generalised Least Squares) parameter estimates with corresponding standard errors are shown, ² following the effect size denotes the effect of the quadratic term. P values are reported as follows: NS, not significant; *p < 0.05; **p < 0.01; ***p < 0.001.

### N-fixer community composition

Total N-fixer community composition was weakly (dbRDA model R^2^ = 0.04) correlated with the bioclimatic variables, the non-N-fixing prokaryotic community, latitude, soil pH, total N and other macroelement content, and the richness of N-fixing plants (**Table S5**). The community composition of rhizobia was weakly correlated with the bioclimatic variables, the non-N-fixing prokaryotic community, latitude, soil pH and total N and other macroelement content, as well as N-fixing plant richness and the historical biome stability in the *nif*H dataset (dbRDA model R^2^ = 0.03, **Table S5**). In the 16S SSU dataset, rhizobial community composition was correlated with soil P but had no correlations with latitude, other soil macroelements, N-fixing plant richness or the historical stability of the biome (dbRDA model R2 = 0.19; **Table S5**). In the *nif*H dataset, Cyanobacterial communities (dbRDA model R^2^ = 0.03) were weakly associated with soil pH, stable hot climate, seasonal humidity and other soil macroelements (**Table S5**), but associated with the non-N-fixing prokaryotic community, latitude, soil pH, P and total N, and stable hot and warm arid climates in the 16S SSU dataset (dbRDA model R^2^ = 0.05, **Table S5**).

## Discussion

The soil microbial component constitutes the base for terrestrial ecosystem functioning. In particular, biological nitrogen fixation, conducted by specific groups of bacteria, is a crucial component of the terrestrial carbon cycle, providing the nitrogen input into ecosystems that plants require. The extent and global distribution of nitrogen fixation are highly disputed. In their meta-analysis, Davies-Barnard & Friedlingstein (2020) found no evidence for any statistically significant relationship between biological nitrogen fixation and conventionally used climatic and soil parameters. However, the lack of this relationship can partly mirror the limited information about the distribution of N-fixing organisms in general. Here we provide the first estimation of the global distribution of N-fixing bacteria in soil that can serve as background information for further estimating the actual potential of biological N-fixation globally and regionally, as well as in the context of particular ecological conditions.

There is ample information about the distribution and diversity of plants hosting symbiotic nitrogen-fixing bacteria (Sprent et al., 2017; Tedersoo et al., 2018; Ardley & Sprent, 2021; Tamme et al., 2021). Given the recorded correlation between plant and bacterial diversity (Porazinska et al., 2018), we expected that large-scale community patterns of N-fixing bacteria to a certain degree mirror those of their host plants. Indeed, we recorded a degree of similarity. For instance, rhizobia richness was positively correlated to warm and moderately arid climates, with a similar pattern being found for the relative richness of rhizobia-associated plants globally (Tamme et al., 2021) and regionally (Pellegrini et al., 2016). Diazotrophic prokaryotes in general have been also shown to be largely limited by aridity (Zhao et al., 2020), but in this study, only rhizobial richness showed a relationship with the bioclimatic principal component describing aridity. Interestingly, Cyanobacteria-associated plants in Tamme et al. (2021) exhibited similar patterns to rhizobia-associated plants, but Cyanobacteria examined in this study responded negatively to higher temperatures. This decoupling is most likely caused by the fact that the *nif*H gene sequencing used in this study enabled to detect a large number of Cyanobacteria that are free-living or in symbiosis with other organism groups, such as fungi in cyanolichens (Rikkinen, 2015), termites (Yamada et al., 2007) and various protists (Nowack & Melkonian, 2010). However, the direct comparison of N-fixing plant richness and the richness of N-fixing bacteria yielded no significant associations. Considering that the larger groups of microbial N-fixing bacteria (i.e. total N-fixers and rhizobia) in this study corresponded similarly to the bioclimatic factors that have shown to influence the richness of N-fixing plants (Tamme et al., 2021), the effect of N-fixing plants on N-fixing bacteria can be masked by the environmental co-variation. In addition, the density of observations in the GBIF data used for assessment of N-fixing plant richness varies significantly at the global scale, with much more records in certain regions, such as Europe and North America, thus also possibly contributing to the lack of a direct correlation between N-fixing plant and microbial richness.

Regional biogeographic history can explain some patterns in the diversity of plants and animals (Mittelbach et al., 2007), but it is rarely considered in microbial ecology. Our study revealed that the biogeographic stability of the sampled region is positively associated with the diversity and abundance of N-fixing bacteria, with regions where the biome has been historically stable since the Last Glacial Maximum hosting a greater richness and Shannon diversity. An analogous relationship was described for arbuscular mycorrhizal fungal communities by Pärtel et al. (2017), who recorded the highest fungal diversity in historically stable tropical grasslands.

Our study revealed that total N-fixer, rhizobial and Cyanobacterial diversity correlated significantly with local non-N-fixing bacterial community composition, highlighting the role of biotic interactions in structuring local microbial communities (Soliveres et al., 2018). Interestingly, rhizobial diversity correlated with the non-N-fixing community composition gradient distinctly from total N-fixing bacteria and Cyanobacteria. These distinct associations between the richness of different groups of N-fixing prokaryotes with the composition of non-N-fixing bacterial community may reflect different levels of dependence on plant hosts within these groups. However, these relationships may also arise from distinct affinities of different microbial groups to environmental factors, and hence the possible causality of the relationships merits further scrutiny. Nevertheless, the often-neglected impact of biotic interactions among bacteria should be considered when addressing the large-scale patterns of N-fixing bacterial communities. Also, further study into the causality and directionality of belowground biotic interactions is direly needed.

The results of this study indicated that the diversity and relative abundance of total N-fixing bacteria and rhizobia are unimodally related to soil pH. At the same time, there is a U-shaped relationship between the diversity and abundance of Cyanobacteria and soil pH. While generally, bacteria exhibit a positive relationship with soil pH (Lauber et al., 2009; Bahram et al., 2018; Delgado-Baquerizo et al., 2018), unimodal relationships are not uncommon (Fierer & Jackson, 2006). However, a U-shape relationship is unexpected. The maximum diversity of Cyanobacteria at high pH corresponds to the general pattern found for bacteria (Bahram et al., 2018). At the same time, the maximum at low pH may reflect the abundant distribution of important host plants of Cyanobacteria – feather mosses – in the boreal zone (DeLuca et al., 2007). Alternatively, the response of Cyanobacteria resembles the realised niche of an inferior competitor, similar to, e.g. *Pinus sylvestris* or Saccharomycetales yeasts (Tedersoo et al., 2020), whose abundance or richness maxima also lie at the extremes of the soil pH gradient. Although in the *nif*H dataset, rhizobial richness in particular was not significantly correlated with soil nitrogen content, in general, the diversity and richness of both total N-fixers and rhizobia across the two amplicons studied were highest at intermediate soil nitrogen contents. Meanwhile, the richness and diversity of Cyanobacteria exhibited a negative correlation with soil nitrogen content. These results are consistent with fertilisation experiments, where nitrogen addition decreased the diversity and abundance of N-fixing bacteria (Wang et al., 2017). At the same time, the positive association between soil P content and the richness and diversity of N-fixing bacteria that could also be expected from previous experiments (Wang et al., 2017) was only demonstrated for Cyanobacteria. This suggests that N is the most important soil nutrient for structuring the composition of N-fixing bacteria.

Overall, global variation in the taxonomic composition of the rhizobial community was driven by multiple climatic, geographic, soil, and biotic factors. Although with fewer significant relationships, similar patterns were revealed for Cyanobacteria. Temperature and soil pH were the strongest drivers of the composition of rhizobial and Cyanobacteria communities, agreeing with the patterns recorded for soil bacteria in general (Fierer & Jackson, 2006; Bahram et al., 2018; Karimi et al., 2020). At the same time, this is the first study demonstrating the importance of biotic drivers, such as the composition of other prokaryotes (on total N-fixers, rhizobia, and Cyanobacteria) and the plant community (on total N-fixers) in shaping the composition of N-fixing bacteria. While the diversity of N-fixers was correlated with the stability of the biome, the community composition remained largely unaffected (except for a very weak correlation in rhizobia). It might be that environmental parameters are mainly driving the communities, and the historical stability of the environment has allowed for more diversification and hence greater richness without selecting specific indicator taxa for stability.

To conclude, we reported a comprehensive evaluation of the global trends of terrestrial nitrogen fixation *via* amplification of the *nif*H gene, supplemented by parallel analysis of similar N-fixer groups in a general 16S SSU prokaryotic dataset. The richness, diversity, relative abundance and community composition of N-fixing prokaryotes are driven mainly by bioclimatic variables, but notably also soil pH. We also demonstrate the interplay of the co-occurring non-N-fixing prokaryotic community composition with the N-fixing community in both composition and diversity. Further research is needed to identify the biotic drivers of N-fixing bacterial communities, e.g. by developing better abilities to distinguish free-living and symbiotic groups of N-fixing bacteria or by elaborating on the potentially facultative nature of N-fixing symbioses.

## Data availability statement

The datasets presented in this study can be found in online repositories. The names of the repository/repositories and accession number(s) can be found below: https://www.ncbi.nlm.nih.gov/, BioProject PRJNA659159https://data.mendeley.com/datasets/dsvcw24cyc/1, doi: 10.17632/dsvcw24cyc.1.

## Author contributions

Conceptualisation, MZ and S-KS. methodology, S-KS, MV, JD, JO. software, MV. formal analysis, S-KS, MV. investigation, MM, MÖ, MZ, MP. resources, SA-Q, MZ, LT. data curation, SA, MB, CB, JC, EF, GD, RD, LF, RG-O, IH, KK, UK, MS, TV, LM, SP, AV-P. writing—original draft preparation, S-KS, MZ, MV. writing—review and editing, JD, MÖ. visualisation, S-KS, MV. project administration, MZ, LT, SA-Q, MM. All authors contributed to the article and approved the submitted version.
