# Supplementary material for: Global diversity and distribution of nitrogen-fixing bacteria in the soil
Source: Front Plant Sci. 2023 Jan 20;14:1100235. doi: 10.3389/fpls.2023.1100235 (PMC9895822; doi:10.3389/fpls.2023.1100235)
Supplement: Supplementary file 1 [file DataSheet_1.docx]

Supplementary Material


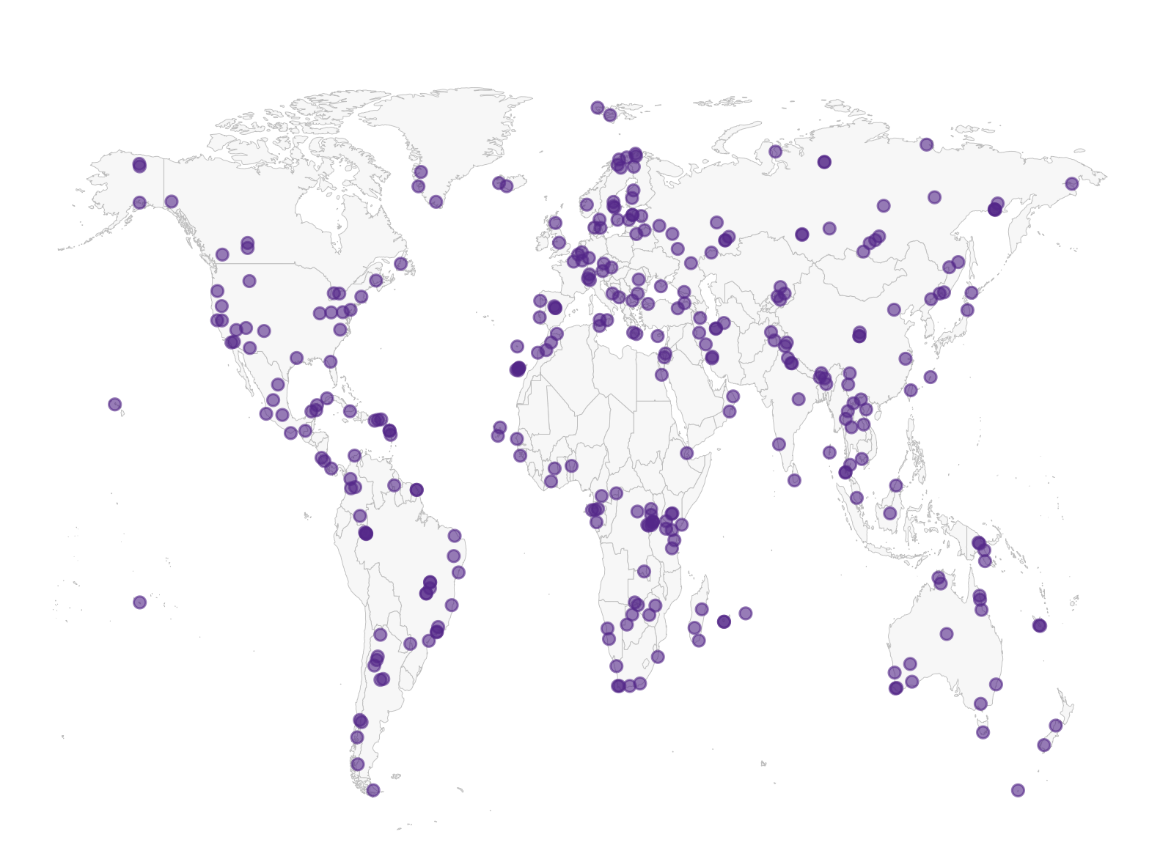


**Figure S1** The global distribution of the sampling points.


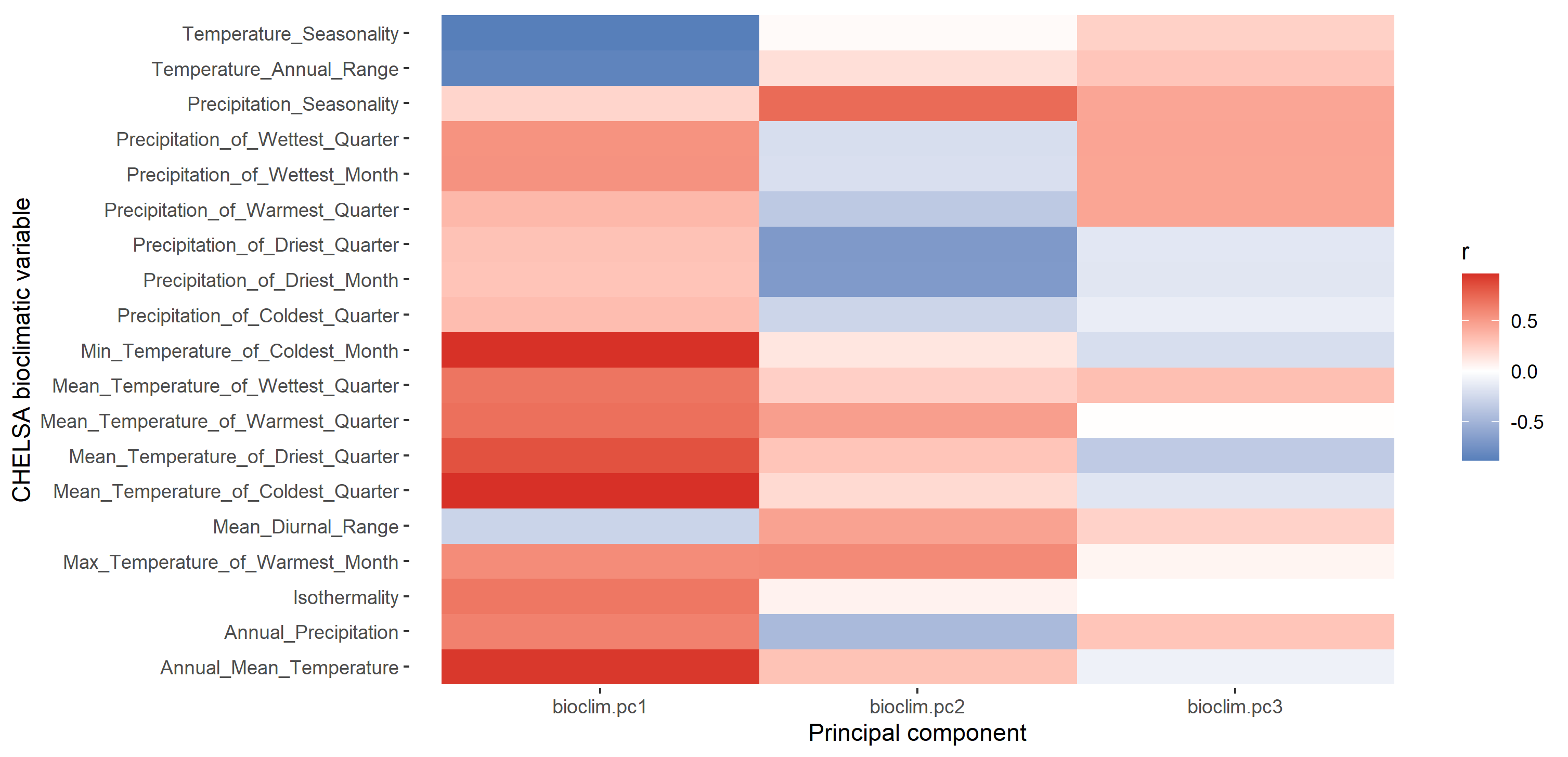


**Figure S2** Correlations of the bioclimatic variables extracted from CHELSA database with the first three principal components incorporated in statistical analyses.


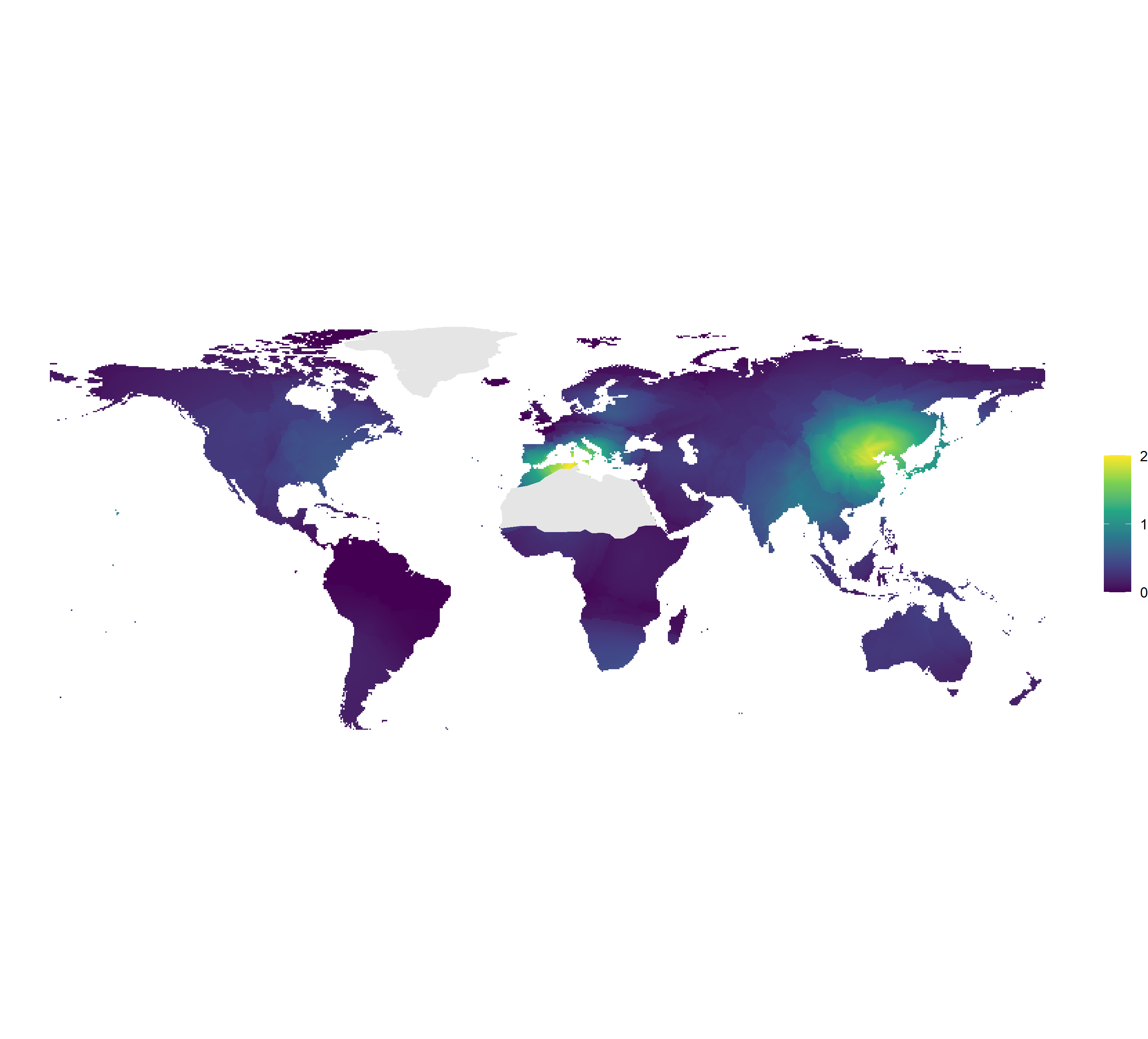


**Figure S3** Interpolated (k-nearest-neighbour map cell interpolation based on the values from the collected samples, cell size = 0.5°×0.5°, k = 18) richness map of Frankia, based on the *nif*H sequencing dataset.

**
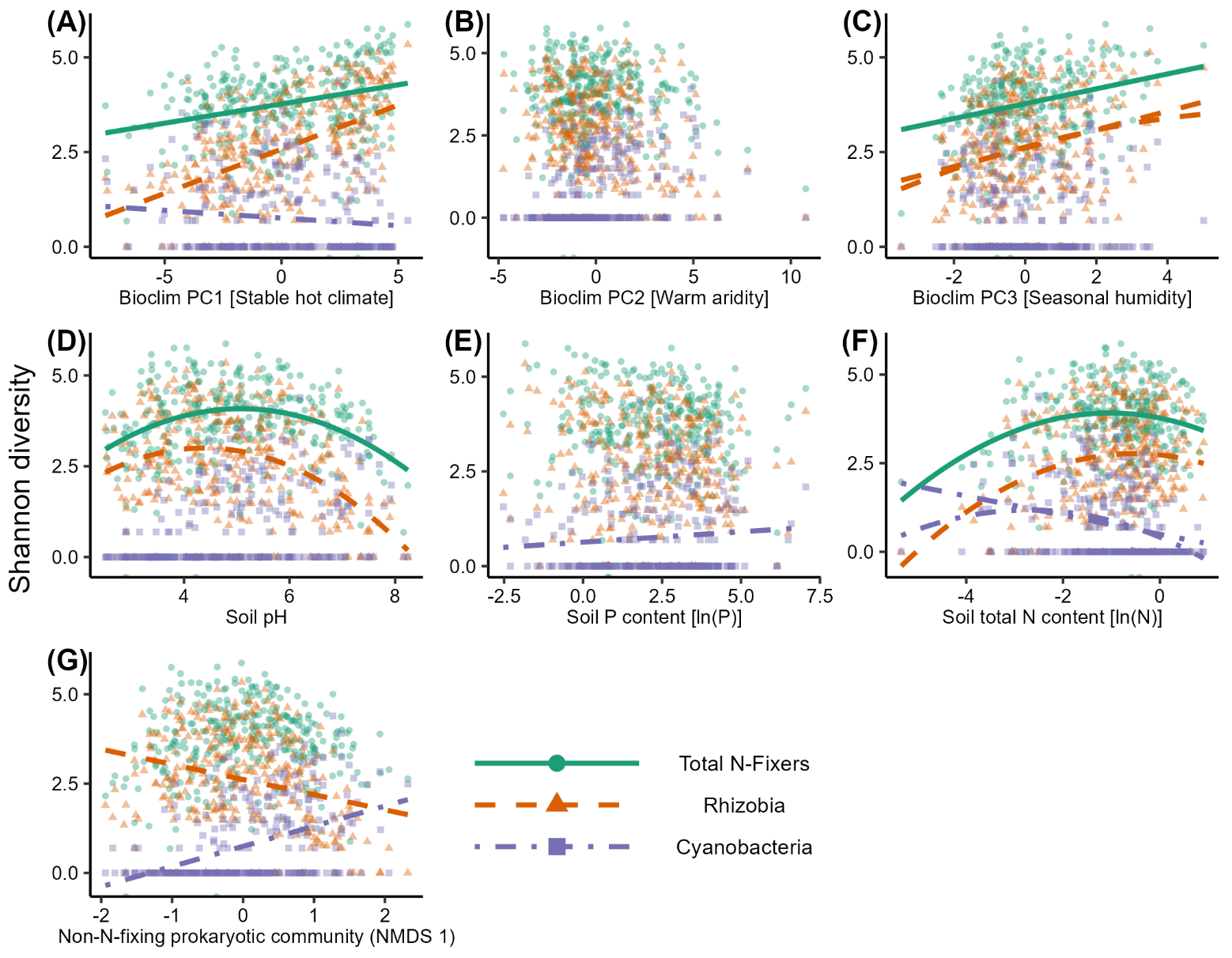
**

**Figure S4** Factors affecting the Shannon diversity index of different groups of N-fixing prokaryotes in soil samples in the nitrogenase reductase *nif*H gene dataset. Lines show predicted values from the GLS model.


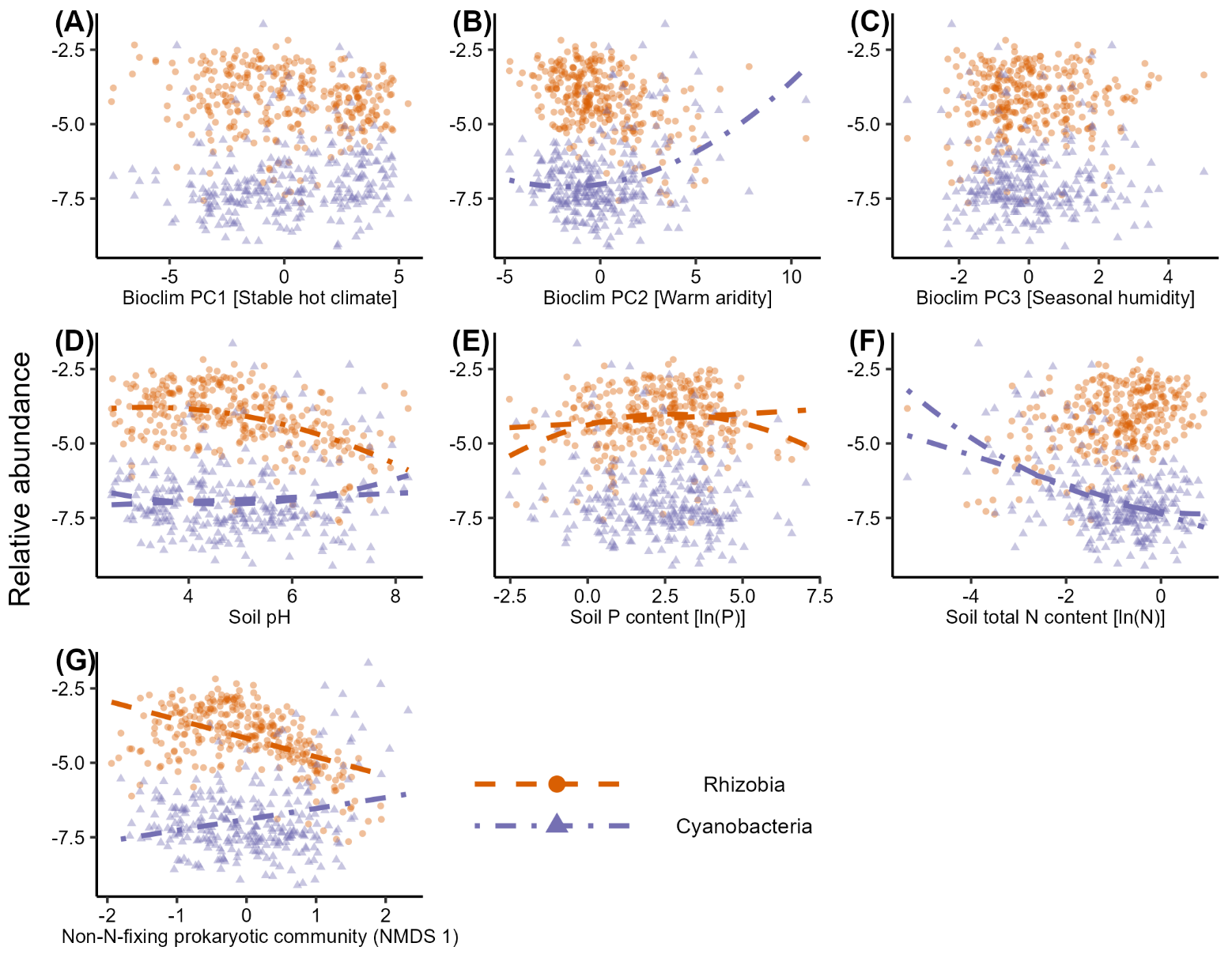


**Figure S5** Factors affecting the relative abundance [ln(abundance of group/(abundance of all prokaryotes – abundance of group))] of different groups of N-fixing prokaryotes in soil samples in the 16S SSU gene dataset. Lines show predicted values from the GLS model.

**Table S1** Habitat characterizations of the sampling locations, sorted by latitude. The biome classification of Olson et al. 2001 [doi:10.1641/0006-3568(2001)051[0933:TEOTWA]2.0.CO;2] is also shown.

|  | **Latitude** | **Longitude** | **Habitat** | **Biome (Olson)** |
| --- | --- | --- | --- | --- |
| 1 | 79.7 | 11.3 | tundra | Tundra |
| 2 | 78.2 | 15.7 | tundra | Tundra |
| 3 | 72.4 | 126.8 | tundra | Tundra |
| 4 | 71.0 | 73.8 | shrubland | Tundra |
| 5 | 70.6 | 24.6 | grassland | Tundra |
| 6 | 70.1 | 24.8 | subpolar coniferous forest | Tundra |
| 7 | 69.9 | 21.6 | subpolar coniferous forest | Tundra |
| 8 | 69.5 | 18.9 | subpolar coniferous forest | Tundra |
| 9 | 69.0 | 91.0 | birch forest | Tundra |
| 10 | 69.0 | 90.9 | shrubland | Tundra |
| 11 | 68.6 | -149.6 | tundra | Tundra |
| 12 | 68.4 | 18.3 | wooded tundra | Tundra |
| 13 | 68.1 | -149.5 | tundra | Tundra |
| 14 | 68.1 | 24.0 | subpolar coniferous forest | Boreal Forests/Taiga |
| 15 | 67.9 | 19.5 | subpolar coniferous forest | Tundra |
| 16 | 67.0 | -50.7 | tundra | Tundra |
| 17 | 64.9 | -23.3 | natural grassland | Boreal Forests/Taiga |
| 18 | 64.7 | 177.9 | tundra | Tundra |
| 19 | 64.2 | -20.6 | tundra | Boreal Forests/Taiga |
| 20 | 64.2 | -51.6 | tundra | Tundra |
| 21 | 63.4 | 23.9 | tundra | Boreal Forests/Taiga |
| 22 | 62.1 | 129.6 | taiga pine forest | Boreal Forests/Taiga |
| 23 | 61.9 | 23.4 | temperate coniferous forest | Boreal Forests/Taiga |
| 24 | 61.2 | -138.3 | tundra | Boreal Forests/Taiga |
| 25 | 61.2 | -45.4 | tundra | Tundra |
| 26 | 61.1 | 16.9 | subpolar coniferous forest | Boreal Forests/Taiga |
| 27 | 61.0 | -149.5 | temperate broadleaf forest | Tundra |
| 28 | 60.8 | 151.8 | tundra | Boreal Forests/Taiga |
| 29 | 60.6 | 7.6 | tundra | Tundra |
| 30 | 60.4 | 111.8 | boreal forest | Boreal Forests/Taiga |
| 31 | 60.3 | 16.9 | boreal forest | Temperate Broadleaf & Mixed Forests |
| 32 | 60.0 | 17.3 | pasture | Temperate Broadleaf & Mixed Forests |
| 33 | 59.7 | 150.7 | forest tundra | Boreal Forests/Taiga |
| 34 | 59.7 | 150.7 | boreal forest | Boreal Forests/Taiga |
| 35 | 59.6 | 150.9 | subpolar coniferous forest | Boreal Forests/Taiga |
| 36 | 58.6 | 23.6 | deciduous forest | Temperate Broadleaf & Mixed Forests |
| 37 | 58.6 | 23.6 | wooded meadow | Temperate Broadleaf & Mixed Forests |
| 38 | 58.4 | 26.7 | temperate grassland | Temperate Grasslands. Savannas & Shrublands |
| 39 | 57.7 | 12.0 | temperate broadleaf forest | Temperate Broadleaf & Mixed Forests |
| 40 | 57.6 | 22.3 | temperate broadleaf forest | Temperate Broadleaf & Mixed Forests |
| 41 | 57.6 | 18.3 | urban | Temperate Broadleaf & Mixed Forests |
| 42 | 57.1 | 53.2 | subpolar coniferous forest | Temperate Broadleaf & Mixed Forests |
| 43 | 57.0 | -3.5 | temperate coniferous forest | Temperate Conifer Forests |
| 44 | 56.5 | 33.0 | temperate coniferous forest | Temperate Broadleaf & Mixed Forests |
| 45 | 56.1 | 10.2 | deciduous forest | Temperate Broadleaf & Mixed Forests |
| 46 | 56.0 | 12.3 | temperate broadleaf forest | Temperate Broadleaf & Mixed Forests |
| 47 | 55.9 | 92.7 | temperate mixed forest | Boreal Forests/Taiga |
| 48 | 55.6 | 27.8 | temperate broadleaf forest | Temperate Broadleaf & Mixed Forests |
| 49 | 54.9 | 37.6 | boreal forest | Temperate Broadleaf & Mixed Forests |
| 50 | 54.8 | 24.9 | oak forest | Temperate Broadleaf & Mixed Forests |
| 51 | 54.8 | 83.1 | aspen forest | Temperate Grasslands. Savannas & Shrublands |
| 52 | 54.7 | 83.1 | meadow-steppe | Temperate Grasslands. Savannas & Shrublands |
| 53 | 54.4 | 110.1 | temperate mixed forest | Boreal Forests/Taiga |
| 54 | 54.3 | 57.4 | mountain coniferous forest | Boreal Forests/Taiga |
| 55 | 53.6 | 108.8 | montane shrubland | Boreal Forests/Taiga |
| 56 | 53.6 | 56.1 | dry Oak forest | Temperate Broadleaf & Mixed Forests |
| 57 | 53.6 | 56.1 | steppe | Temperate Broadleaf & Mixed Forests |
| 58 | 53.1 | -2.2 | temperate broadleaf forest | Temperate Broadleaf & Mixed Forests |
| 59 | 53.1 | -111.6 | prairie | Temperate Grasslands. Savannas & Shrublands |
| 60 | 53.0 | 106.9 | steppe | Boreal Forests/Taiga |
| 61 | 52.1 | -111.6 | prairie | Temperate Grasslands. Savannas & Shrublands |
| 62 | 51.9 | 39.5 | temperate coniferous forest | Temperate Broadleaf & Mixed Forests |
| 63 | 51.4 | 104.7 | hemiboreal forest | Temperate Conifer Forests |
| 64 | 51.3 | 5.8 | temperate broadleaf forest | Temperate Broadleaf & Mixed Forests |
| 65 | 51.2 | 51.3 | steppe | Temperate Grasslands. Savannas & Shrublands |
| 66 | 50.8 | 4.5 | temperate broadleaf forest | Temperate Broadleaf & Mixed Forests |
| 67 | 50.8 | -120.4 | upper grassland | Temperate Conifer Forests |
| 68 | 50.1 | 8.3 | temperate broadleaf forest | Temperate Broadleaf & Mixed Forests |
| 69 | 49.6 | 5.9 | temperate broadleaf forest | Temperate Broadleaf & Mixed Forests |
| 70 | 49.4 | 2.9 | deciduous forest | Temperate Broadleaf & Mixed Forests |
| 71 | 49.3 | 137.9 | temperate mixed forest | Boreal Forests/Taiga |
| 72 | 49.1 | 44.1 | steppe | Temperate Grasslands. Savannas & Shrublands |
| 73 | 49.0 | 13.5 | subpolar coniferous forest | Temperate Broadleaf & Mixed Forests |
| 74 | 49.0 | -57.8 | temperate broadleaf forest | Boreal Forests/Taiga |
| 75 | 48.3 | 134.8 | temperate broadleaf forest | Flooded Grasslands & Savannas |
| 76 | 48.3 | 16.2 | temperate broadleaf & mixed forests | Temperate Broadleaf & Mixed Forests |
| 77 | 47.6 | 13.1 | montane grassland | Temperate Conifer Forests |
| 78 | 46.9 | 8.5 | montane grassland | Temperate Conifer Forests |
| 79 | 46.1 | 7.9 | temperate coniferous forest | Temperate Conifer Forests |
| 80 | 45.9 | 25.7 | temperate broadleaf forest | Temperate Broadleaf & Mixed Forests |
| 81 | 45.8 | 8.6 | temperate broadleaf forest | Temperate Conifer Forests |
| 82 | 45.7 | -66.5 | temperate broadleaf & mixed forest | Temperate Broadleaf & Mixed Forests |
| 83 | 45.6 | -110.9 | subalpine temperate coniferous forest | Temperate Conifer Forests |
| 84 | 44.6 | 33.5 | steppe | Temperate Broadleaf & Mixed Forests |
| 85 | 44.4 | 75.5 | dune sand desert | Deserts & Xeric Shrublands |
| 86 | 43.6 | -122.2 | temperate coniferous forest | Temperate Conifer Forests |
| 87 | 43.4 | 41.7 | subalpine shrub | Temperate Broadleaf & Mixed Forests |
| 88 | 43.4 | 132.9 | subpolar coniferous forest | Temperate Broadleaf & Mixed Forests |
| 89 | 43.3 | 142.6 | subpolar coniferous forest | Temperate Conifer Forests |
| 90 | 43.2 | -81.3 | grassland | Temperate Broadleaf & Mixed Forests |
| 91 | 43.1 | 16.5 | temperate coniferous forest | Mediterranean Forests. Woodlands & Scrub |
| 92 | 43.1 | 25.4 | temperate broadleaf forest | Temperate Broadleaf & Mixed Forests |
| 93 | 43.1 | -79.4 | temperate broadleaf forest | Temperate Broadleaf & Mixed Forests |
| 94 | 43.1 | 77.0 | temperate coniferous forest | Montane Grasslands & Shrublands |
| 95 | 43.1 | 131.4 | temperate broadleaf forest | Temperate Broadleaf & Mixed Forests |
| 96 | 42.6 | 74.5 | montane shrubland | Temperate Conifer Forests |
| 97 | 42.5 | -71.6 | temperate broadleaf forest | Temperate Broadleaf & Mixed Forests |
| 98 | 42.4 | 18.8 | temperate broadleaf forest | Mediterranean Forests. Woodlands & Scrub |
| 99 | 42.0 | 128.4 | subpolar coniferous forest | Temperate Broadleaf & Mixed Forests |
| 100 | 41.9 | 75.4 | montane grassland | Montane Grasslands & Shrublands |
| 101 | 41.8 | 23.5 | temperate mixed forest | Temperate Broadleaf & Mixed Forests |
| 102 | 41.7 | -8.8 | temperate mixed forest | Temperate Broadleaf & Mixed Forests |
| 103 | 41.2 | 41.8 | temperate broadleaf forest | Temperate Broadleaf & Mixed Forests |
| 104 | 41.1 | 29.1 | temperate woodland | Temperate Broadleaf & Mixed Forests |
| 105 | 40.7 | -120.7 | flooded grassland | Temperate Conifer Forests |
| 106 | 40.6 | -3.9 | oak forest | Mediterranean Forests. Woodlands & Scrub |
| 107 | 40.3 | -3.5 | semiarid grassland | Mediterranean Forests. Woodlands & Scrub |
| 108 | 40.3 | -3.5 | mediterranean garrigue | Mediterranean Forests. Woodlands & Scrub |
| 109 | 40.2 | 39.5 | temperate shrubland | Temperate Conifer Forests |
| 110 | 40.0 | 115.4 | subpolar coniferous forest | Temperate Broadleaf & Mixed Forests |
| 111 | 39.9 | -75.4 | decidious forest | Temperate Broadleaf & Mixed Forests |
| 112 | 39.9 | 141.2 | temperate broadleaf forest | Temperate Broadleaf & Mixed Forests |
| 113 | 39.5 | -78.2 | temperate broadleaf forest | Temperate Broadleaf & Mixed Forests |
| 114 | 39.4 | -82.2 | tropical broadleaf forest | Temperate Broadleaf & Mixed Forests |
| 115 | 39.2 | -86.2 | old growth deciduous forest. nature reserve | Temperate Broadleaf & Mixed Forests |
| 116 | 38.5 | -9.0 | temperate broadleaf forest | Mediterranean Forests. Woodlands & Scrub |
| 117 | 38.3 | 47.3 | subtropical broadleaf forest | Temperate Conifer Forests |
| 118 | 38.0 | 12.1 | temperate shrubland | Mediterranean Forests. Woodlands & Scrub |
| 119 | 37.9 | 14.7 | temperate broadleaf forest | Mediterranean Forests. Woodlands & Scrub |
| 120 | 37.9 | -122.2 | mediterranean forest | Mediterranean Forests. Woodlands & Scrub |
| 121 | 37.8 | -120.6 | temperate broadleaf forest | Mediterranean Forests. Woodlands & Scrub |
| 122 | 37.4 | 55.7 | temperate broadleaf forest | Temperate Broadleaf & Mixed Forests |
| 123 | 36.8 | 12.0 | subtropical shrubland | Mediterranean Forests. Woodlands & Scrub |
| 124 | 36.4 | -112.1 | temperate grassland | Temperate Conifer Forests |
| 125 | 36.3 | 52.9 | temperate broadleaf forest | Temperate Broadleaf & Mixed Forests |
| 126 | 36.2 | 52.8 | temperate broadleaf forest | Temperate Broadleaf & Mixed Forests |
| 127 | 36.0 | -79.1 | temperate broadleaf forest | Temperate Broadleaf & Mixed Forests |
| 128 | 36.0 | -115.5 | temperate coniferous forest | Deserts & Xeric Shrublands |
| 129 | 35.8 | -105.8 | temperate broadleaf forest | Temperate Conifer Forests |
| 130 | 35.6 | 72.2 | temperate broadleaf forest | Montane Grasslands & Shrublands |
| 131 | 35.5 | 103.3 | seminatural grassland | Montane Grasslands & Shrublands |
| 132 | 35.5 | 23.8 | mediterranean scrub | Mediterranean Forests. Woodlands & Scrub |
| 133 | 35.4 | 47.0 | subtropical desert | Temperate Broadleaf & Mixed Forests |
| 134 | 35.2 | -3.0 | subtropical coniferous forest | Mediterranean Forests. Woodlands & Scrub |
| 135 | 35.2 | 24.9 | mediterranean forest | Mediterranean Forests. Woodlands & Scrub |
| 136 | 34.8 | 32.4 | mediterranean forests. woodlands & scrubs | Mediterranean Forests. Woodlands & Scrub |
| 137 | 34.8 | 103.2 | alpine meadow | Temperate Conifer Forests |
| 138 | 34.7 | 103.2 | alpine meadow | Temperate Conifer Forests |
| 139 | 33.9 | 73.4 | temperate broadleaf forest | Temperate Broadleaf & Mixed Forests |
| 140 | 33.7 | -116.4 | deserts and xeric shrublands | Deserts & Xeric Shrublands |
| 141 | 33.5 | -117.2 | temperate natural grassland | Mediterranean Forests. Woodlands & Scrub |
| 142 | 33.5 | -5.0 | mediterranean forest | Temperate Conifer Forests |
| 143 | 33.5 | 77.8 | montane desert | Montane Grasslands & Shrublands |
| 144 | 33.2 | 49.3 | temperate broadleaf forest | Temperate Broadleaf & Mixed Forests |
| 145 | 32.8 | 77.1 | montane grassland | Montane Grasslands & Shrublands |
| 146 | 32.7 | -16.9 | subtropical broadleaf forest | Temperate Broadleaf & Mixed Forests |
| 147 | 32.4 | -110.8 | temperate coniferous forest | Tropical & Subtropical Coniferous Forests |
| 148 | 32.0 | -6.7 | subtropical woodland | Mediterranean Forests. Woodlands & Scrub |
| 149 | 31.5 | -9.7 | subtropical shrubland | Mediterranean Forests. Woodlands & Scrub |
| 150 | 31.3 | 35.1 | temperate shrubland | Deserts & Xeric Shrublands |
| 151 | 30.7 | 51.6 | tropical broadleaf forest | Temperate Broadleaf & Mixed Forests |
| 152 | 30.6 | 34.7 | deserts and xeric shrublands | Deserts & Xeric Shrublands |
| 153 | 30.5 | -94.4 | subtropical broadleaf forest | Temperate Conifer Forests |
| 154 | 30.5 | 78.1 | temperate broadleaf forest | Tropical & Subtropical Moist Broadleaf Forests |
| 155 | 30.4 | 51.5 | temperate broadleaf forest | Temperate Broadleaf & Mixed Forests |
| 156 | 30.3 | 119.4 | temperate coniferous forest | Temperate Broadleaf & Mixed Forests |
| 157 | 29.7 | -82.4 | subtropical broadleaf forest | Temperate Grasslands. Savannas & Shrublands |
| 158 | 29.5 | 79.2 | tropical broadleaf forest | Tropical & Subtropical Coniferous Forests |
| 159 | 29.4 | 79.6 | deciduous forest | Tropical & Subtropical Coniferous Forests |
| 160 | 28.5 | -16.2 | subtropical broadleaf forest | Mediterranean Forests. Woodlands & Scrub |
| 161 | 28.5 | -16.3 | laurel forest | Mediterranean Forests. Woodlands & Scrub |
| 162 | 28.4 | -16.5 | ericaceous pine forest | Mediterranean Forests. Woodlands & Scrub |
| 163 | 28.1 | -17.3 | subtropical coniferous forest | Mediterranean Forests. Woodlands & Scrub |
| 164 | 28.0 | -16.5 | shrubland | Mediterranean Forests. Woodlands & Scrub |
| 165 | 27.5 | 89.9 | subtropical broadleaf forest | Temperate Broadleaf & Mixed Forests |
| 166 | 27.5 | 99.9 | temperate coniferous forest | Temperate Conifer Forests |
| 167 | 27.2 | 33.8 | subtropical desert | Deserts & Xeric Shrublands |
| 168 | 26.7 | 128.2 | temperate broadleaf forest | Tropical & Subtropical Moist Broadleaf Forests |
| 169 | 26.7 | 89.3 | tropical broadleaf forest | Tropical & Subtropical Moist Broadleaf Forests |
| 170 | 26.5 | 91.1 | tropical grassland | Tropical & Subtropical Moist Broadleaf Forests |
| 171 | 25.4 | 91.6 | tropical broadleaf forest | Tropical & Subtropical Moist Broadleaf Forests |
| 172 | 25.3 | 99.3 | temperate coniferous forest | Tropical & Subtropical Moist Broadleaf Forests |
| 173 | 25.2 | -100.9 | subtropical coniferous forest | Tropical & Subtropical Coniferous Forests |
| 174 | 24.1 | 121.3 | temperate coniferous forest | Tropical & Subtropical Moist Broadleaf Forests |
| 175 | 22.9 | 58.9 | montane desert | Temperate Grasslands. Savannas & Shrublands |
| 176 | 22.5 | -83.7 | tropical broadleaf forest | Tropical & Subtropical Coniferous Forests |
| 177 | 22.4 | 81.9 | deciduous forest | Tropical & Subtropical Moist Broadleaf Forests |
| 178 | 22.3 | 103.8 | tropical broadleaf forest | Tropical & Subtropical Moist Broadleaf Forests |
| 179 | 22.2 | -102.6 | tropical broadleaf forest | Tropical & Subtropical Coniferous Forests |
| 180 | 21.6 | 101.2 | temperate broadleaf forest | Tropical & Subtropical Moist Broadleaf Forests |
| 181 | 21.4 | -158.2 | tropical broadleaf forest | Tropical & Subtropical Moist Broadleaf Forests |
| 182 | 21.2 | -87.2 | tropical broadleaf forest | Tropical & Subtropical Moist Broadleaf Forests |
| 183 | 20.3 | 105.6 | tropical broadleaf forest | Tropical & Subtropical Moist Broadleaf Forests |
| 184 | 20.2 | -87.5 | tropical broadleaf forest | Tropical & Subtropical Moist Broadleaf Forests |
| 185 | 20.0 | 99.2 | tropical coniferous forest | Tropical & Subtropical Moist Broadleaf Forests |
| 186 | 20.0 | -75.6 | tropical broadleaf forest | Tropical & Subtropical Moist Broadleaf Forests |
| 187 | 20.0 | -89.2 | tropical broadleaf forest | Tropical & Subtropical Dry Broadleaf Forests |
| 188 | 20.0 | 57.7 | tropical desert | Deserts & Xeric Shrublands |
| 189 | 19.5 | -105.0 | tropical broadleaf forest | Tropical & Subtropical Dry Broadleaf Forests |
| 190 | 19.3 | -99.4 | tropical broadleaf forest | Tropical & Subtropical Coniferous Forests |
| 191 | 18.5 | 98.4 | tropical broadleaf forest | Tropical & Subtropical Moist Broadleaf Forests |
| 192 | 18.4 | -64.7 | tropical broadleaf forest | Tropical & Subtropical Moist Broadleaf Forests |
| 193 | 18.3 | -65.8 | tropical broadleaf forest | Tropical & Subtropical Moist Broadleaf Forests |
| 194 | 18.1 | -67.0 | tropical broadleaf forest | Tropical & Subtropical Moist Broadleaf Forests |
| 195 | 17.4 | 104.8 | tropical moist broadleaf forest | Tropical & Subtropical Dry Broadleaf Forests |
| 196 | 16.9 | 100.5 | seasonal tropical forest | Tropical & Subtropical Dry Broadleaf Forests |
| 197 | 16.8 | -22.9 | semidesert | Tropical & Subtropical Dry Broadleaf Forests |
| 198 | 16.2 | -61.7 | tropical broadleaf forest | Tropical & Subtropical Moist Broadleaf Forests |
| 199 | 16.2 | -61.7 | tropical rainforest | Tropical & Subtropical Moist Broadleaf Forests |
| 200 | 16.2 | -91.3 | tropical broadleaf forest | Tropical & Subtropical Moist Broadleaf Forests |
| 201 | 15.7 | -96.4 | tropical broadleaf forest | Tropical & Subtropical Dry Broadleaf Forests |
| 202 | 15.4 | -61.3 | tropical broadleaf forest | Tropical & Subtropical Moist Broadleaf Forests |
| 203 | 15.2 | -23.7 | arid montane shrubland | Tropical & Subtropical Dry Broadleaf Forests |
| 204 | 14.6 | -17.0 | savannah | Tropical & Subtropical Grasslands. Savannas & Shrublands |
| 205 | 13.5 | 75.0 | tropical broadleaf forest | Tropical & Subtropical Moist Broadleaf Forests |
| 206 | 11.9 | 92.8 | tropical broadleaf forest | Tropical & Subtropical Moist Broadleaf Forests |
| 207 | 11.8 | 42.7 | forest | Deserts & Xeric Shrublands |
| 208 | 11.3 | -74.0 | tropical woodland | Deserts & Xeric Shrublands |
| 209 | 11.3 | -15.8 | tropical rainforest | Tropical & Subtropical Grasslands. Savannas & Shrublands |
| 210 | 10.8 | -85.6 | deciduous forest | Tropical & Subtropical Dry Broadleaf Forests |
| 211 | 10.6 | 104.1 | mountain tropical forest | Tropical & Subtropical Moist Broadleaf Forests |
| 212 | 10.2 | -84.6 | tropical broadleaf forest | Tropical & Subtropical Moist Broadleaf Forests |
| 213 | 9.5 | 100.0 | tropical broadleaf forest | Tropical & Subtropical Moist Broadleaf Forests |
| 214 | 9.3 | 2.2 | tropical woodland | Tropical & Subtropical Grasslands. Savannas & Shrublands |
| 215 | 8.8 | -3.8 | tropical woodland | Tropical & Subtropical Grasslands. Savannas & Shrublands |
| 216 | 8.8 | -82.2 | tropical broadleaf forest | Tropical & Subtropical Moist Broadleaf Forests |
| 217 | 8.0 | 98.4 | seasonal tropical broadleaved forest | Tropical & Subtropical Moist Broadleaf Forests |
| 218 | 7.9 | 98.3 | seasonal tropical broadleaved forest | Tropical & Subtropical Moist Broadleaf Forests |
| 219 | 6.7 | -75.5 | tropical moist broadleaf forest | Tropical & Subtropical Moist Broadleaf Forests |
| 220 | 6.4 | 80.4 | tropical broadleaf forest | Tropical & Subtropical Moist Broadleaf Forests |
| 221 | 6.2 | -5.0 | tropical grasslands & savannas | Tropical & Subtropical Moist Broadleaf Forests |
| 222 | 5.4 | -60.1 | tropical broadleaf forest | Tropical & Subtropical Moist Broadleaf Forests |
| 223 | 5.4 | 116.1 | tropical broadleaf forest | Tropical & Subtropical Moist Broadleaf Forests |
| 224 | 5.1 | -73.8 | high Andean secondary dry tropic forest | Tropical & Subtropical Moist Broadleaf Forests |
| 225 | 4.9 | -75.3 | montane shrubland | Tropical & Subtropical Moist Broadleaf Forests |
| 226 | 4.6 | -52.2 | tropical rainforest | Tropical & Subtropical Moist Broadleaf Forests |
| 227 | 4.6 | -52.2 | tropical broadleaf forest | Tropical & Subtropical Moist Broadleaf Forests |
| 228 | 3.9 | 17.9 | semi-deciduous forest | Tropical & Subtropical Moist Broadleaf Forests |
| 229 | 3.4 | 12.7 | tropical broadleaf forest | Tropical & Subtropical Moist Broadleaf Forests |
| 230 | 3.0 | 102.3 | tropical broadleaf forest | Tropical & Subtropical Moist Broadleaf Forests |
| 231 | 0.8 | 11.5 | tropical broadleaf forest | Tropical & Subtropical Moist Broadleaf Forests |
| 232 | 0.8 | 30.1 | tropical broadleaf forest | Tropical & Subtropical Grasslands. Savannas & Shrublands |
| 233 | 0.6 | 10.4 | tropical rainforest | Tropical & Subtropical Moist Broadleaf Forests |
| 234 | 0.6 | 9.3 | tropical broadleaf forest | Tropical & Subtropical Moist Broadleaf Forests |
| 235 | 0.3 | 25.3 | tropical broadleaf forest | Tropical & Subtropical Moist Broadleaf Forests |
| 236 | -0.1 | 37.6 | mountain forest | Tropical & Subtropical Moist Broadleaf Forests |
| 237 | -0.1 | 114.0 | tropical broadleaf forest | Tropical & Subtropical Moist Broadleaf Forests |
| 238 | -0.2 | 37.3 | alpine Dendrosenecio | Montane Grasslands & Shrublands |
| 239 | -0.4 | 30.1 | tropical broadleaf forest | Tropical & Subtropical Moist Broadleaf Forests |
| 240 | -0.6 | -72.1 | tropical moist broadleaf forest | Tropical & Subtropical Moist Broadleaf Forests |
| 241 | -1.6 | 35.4 | tropical woodland | Tropical & Subtropical Grasslands. Savannas & Shrublands |
| 242 | -1.7 | 30.7 | tropical savanna grassland | Tropical & Subtropical Grasslands. Savannas & Shrublands |
| 243 | -1.7 | 30.7 | tropical woodland | Tropical & Subtropical Grasslands. Savannas & Shrublands |
| 244 | -1.8 | 10.9 | tropical grassland | Tropical & Subtropical Grasslands. Savannas & Shrublands |
| 245 | -1.9 | 30.7 | tropical savanna grassland | Tropical & Subtropical Grasslands. Savannas & Shrublands |
| 246 | -2.3 | 40.9 | tropical grassland | Tropical & Subtropical Moist Broadleaf Forests |
| 247 | -2.3 | 28.8 | tropical broadleaf forest | Tropical & Subtropical Moist Broadleaf Forests |
| 248 | -2.4 | 30.2 | tropical broadleaf forest | Tropical & Subtropical Grasslands. Savannas & Shrublands |
| 249 | -2.5 | 29.2 | rainforest | Tropical & Subtropical Moist Broadleaf Forests |
| 250 | -3.1 | 35.4 | tropical grassland | Tropical & Subtropical Grasslands. Savannas & Shrublands |
| 251 | -3.4 | 37.4 | tropical broadleaf forest | Tropical & Subtropical Grasslands. Savannas & Shrublands |
| 252 | -3.8 | -70.4 | secondary forest | Tropical & Subtropical Moist Broadleaf Forests |
| 253 | -4.0 | -69.9 | tropical moist broadleaf forest | Tropical & Subtropical Moist Broadleaf Forests |
| 254 | -4.0 | -70.0 | lowland tropical rainforest | Tropical & Subtropical Moist Broadleaf Forests |
| 255 | -4.1 | -69.9 | lowland tropical rainforest | Tropical & Subtropical Moist Broadleaf Forests |
| 256 | -4.5 | -38.9 | tropical broadleaf forest | Tropical & Subtropical Dry Broadleaf Forests |
| 257 | -5.3 | 38.3 | tropical broadleaf forest | Tropical & Subtropical Grasslands. Savannas & Shrublands |
| 258 | -5.8 | 145.1 | montane shrubland | Montane Grasslands & Shrublands |
| 259 | -6.0 | 145.4 | tropical broadleaf forest | Tropical & Subtropical Moist Broadleaf Forests |
| 260 | -6.9 | 37.4 | tropical woodland | Flooded Grasslands & Savannas |
| 261 | -7.3 | 147.1 | tropical broadleaf forest | Tropical & Subtropical Moist Broadleaf Forests |
| 262 | -8.5 | -39.2 | flooded grassland | Tropical & Subtropical Dry Broadleaf Forests |
| 263 | -9.4 | 147.3 | tropical broadleaf forest | Tropical & Subtropical Moist Broadleaf Forests |
| 264 | -11.5 | 27.7 | tropical woodland | Tropical & Subtropical Grasslands. Savannas & Shrublands |
| 265 | -11.7 | -37.5 | tropical broadleaf forest | Tropical & Subtropical Moist Broadleaf Forests |
| 266 | -12.7 | 131.0 | tropical broadleaf forest | Tropical & Subtropical Grasslands. Savannas & Shrublands |
| 267 | -13.6 | -47.5 | tropical woodland | Tropical & Subtropical Grasslands. Savannas & Shrublands |
| 268 | -13.6 | -47.5 | tropical woodland | Tropical & Subtropical Grasslands. Savannas & Shrublands |
| 269 | -13.8 | 131.8 | tropical woodland | Tropical & Subtropical Grasslands. Savannas & Shrublands |
| 270 | -14.8 | -47.6 | cerrado | Tropical & Subtropical Grasslands. Savannas & Shrublands |
| 271 | -15.8 | -48.9 | tropical woodland | Tropical & Subtropical Grasslands. Savannas & Shrublands |
| 272 | -15.8 | -48.9 | cerrado | Tropical & Subtropical Grasslands. Savannas & Shrublands |
| 273 | -16.2 | 145.4 | tropical broadleaf forest | Tropical & Subtropical Moist Broadleaf Forests |
| 274 | -17.0 | 145.6 | tropical broadleaf forest | Tropical & Subtropical Moist Broadleaf Forests |
| 275 | -17.6 | -149.4 | wet tropical forest | Tropical & Subtropical Moist Broadleaf Forests |
| 276 | -17.6 | 24.4 | tropical grasslands & savannas | Tropical & Subtropical Grasslands. Savannas & Shrublands |
| 277 | -18.1 | 25.5 | tropical woodland | Tropical & Subtropical Grasslands. Savannas & Shrublands |
| 278 | -18.1 | -39.9 | tropical broadleaf forest | Tropical & Subtropical Moist Broadleaf Forests |
| 279 | -18.2 | 31.6 | tropical woodland | Tropical & Subtropical Grasslands. Savannas & Shrublands |
| 280 | -18.9 | 47.9 | tropical broadleaf forest | Tropical & Subtropical Moist Broadleaf Forests |
| 281 | -19.0 | 146.1 | tropical broadleaf forest | Tropical & Subtropical Moist Broadleaf Forests |
| 282 | -19.7 | 63.3 | tropical broadleaf forest | Tropical & Subtropical Moist Broadleaf Forests |
| 283 | -19.9 | 23.5 | road margin | Tropical & Subtropical Grasslands. Savannas & Shrublands |
| 284 | -20.0 | 29.5 | tropical woodland | Tropical & Subtropical Grasslands. Savannas & Shrublands |
| 285 | -21.4 | 55.7 | tropical broadleaf forest | Tropical & Subtropical Moist Broadleaf Forests |
| 286 | -21.4 | 55.7 | tropical moist broadleaf forest | Tropical & Subtropical Moist Broadleaf Forests |
| 287 | -21.9 | 21.6 | tropical shrubland | Deserts & Xeric Shrublands |
| 288 | -22.1 | 166.3 | tropical broadleaf forest | Tropical & Subtropical Moist Broadleaf Forests |
| 289 | -22.2 | 166.7 | maquis minier | Tropical & Subtropical Moist Broadleaf Forests |
| 290 | -22.4 | -44.7 | montane shrubland | Tropical & Subtropical Grasslands. Savannas & Shrublands |
| 291 | -22.6 | 45.4 | tropical woodland | Deserts & Xeric Shrublands |
| 292 | -22.7 | 14.8 | tropical desert | Deserts & Xeric Shrublands |
| 293 | -23.3 | -45.1 | atlantic forest | Tropical & Subtropical Moist Broadleaf Forests |
| 294 | -23.4 | -45.2 | atlantic forest | Tropical & Subtropical Moist Broadleaf Forests |
| 295 | -23.8 | 133.9 | desert | Deserts & Xeric Shrublands |
| 296 | -23.9 | -64.9 | subtropical broadleaf forest | Tropical & Subtropical Moist Broadleaf Forests |
| 297 | -24.7 | 15.3 | tropical desert | Deserts & Xeric Shrublands |
| 298 | -25.1 | 46.9 | tropical broadleaf forest | Tropical & Subtropical Moist Broadleaf Forests |
| 299 | -25.1 | -47.9 | atlantic forest | Tropical & Subtropical Moist Broadleaf Forests |
| 300 | -25.7 | -54.4 | subtropical broadleaf forest | Tropical & Subtropical Moist Broadleaf Forests |
| 301 | -28.2 | -65.9 | montane Yunga Forest | Tropical & Subtropical Grasslands. Savannas & Shrublands |
| 302 | -28.3 | 32.5 | subtropical broadleaf forest | Tropical & Subtropical Moist Broadleaf Forests |
| 303 | -28.9 | -66.3 | dry Chaco Forest | Tropical & Subtropical Grasslands. Savannas & Shrublands |
| 304 | -29.7 | 121.1 | mulga shrubland | Deserts & Xeric Shrublands |
| 305 | -30.0 | -67.1 | monte desert | Tropical & Subtropical Grasslands. Savannas & Shrublands |
| 306 | -30.1 | 17.9 | desert | Deserts & Xeric Shrublands |
| 307 | -31.4 | 115.6 | woodland | Mediterranean Forests. Woodlands & Scrub |
| 308 | -32.6 | -64.0 | spiny chaco forest | Temperate Grasslands. Savannas & Shrublands |
| 309 | -32.8 | -64.9 | montane pampa grassland | Tropical & Subtropical Grasslands. Savannas & Shrublands |
| 310 | -33.1 | 121.7 | eucalyptus woodland | Mediterranean Forests. Woodlands & Scrub |
| 311 | -33.5 | 26.2 | subtropical shrubland | Mediterranean Forests. Woodlands & Scrub |
| 312 | -33.7 | 151.2 | eucalypt forest | Temperate Broadleaf & Mixed Forests |
| 313 | -34.0 | 22.6 | subtropical broadleaf forest | Tropical & Subtropical Moist Broadleaf Forests |
| 314 | -34.0 | 18.4 | subtropical broadleaf forest | Mediterranean Forests. Woodlands & Scrub |
| 315 | -34.0 | 19.0 | fynbos | Mediterranean Forests. Woodlands & Scrub |
| 316 | -34.4 | 116.3 | tropical broadleaf forest | Mediterranean Forests. Woodlands & Scrub |
| 317 | -34.5 | 115.9 | eucalypt forest | Mediterranean Forests. Woodlands & Scrub |
| 318 | -37.5 | 145.8 | subtropical broadleaf forest | Temperate Broadleaf & Mixed Forests |
| 319 | -40.7 | -72.2 | temperate broadleaf forest | Temperate Broadleaf & Mixed Forests |
| 320 | -41.1 | -71.6 | temperate broadleaf forest | Temperate Broadleaf & Mixed Forests |
| 321 | -41.8 | 172.2 | temperate broadleaf forest | Temperate Broadleaf & Mixed Forests |
| 322 | -43.1 | 146.7 | temperate broadleaf forest | Temperate Broadleaf & Mixed Forests |
| 323 | -44.1 | -73.1 | temperate broadleaf forest | Temperate Broadleaf & Mixed Forests |
| 324 | -45.6 | 168.1 | temperate broadleaf forest | Temperate Broadleaf & Mixed Forests |
| 325 | -49.4 | -72.9 | temperate broadleaf forest | Temperate Broadleaf & Mixed Forests |
| 326 | -54.5 | -67.4 | temperate broadleaf forest | Temperate Broadleaf & Mixed Forests |
| 327 | -54.5 | 158.9 | tundra | Tundra |

**Table S2** Comparison of the biome classification of Olson et al. 2001 [doi:10.1641/0006-3568(2001)051[0933:TEOTWA]2.0.CO;2] and the combined simplified biomes used in the analyses.

| **Olson et al.’s biome** | **Simplified ‘biome’** |
| --- | --- |
| 1 Tropical & Subtropical Moist Broadleaf Forests  2 Tropical & Subtropical Dry Broadleaf Forests  3 Tropical & Subtropical Coniferous Forests | Tropical forests |
| 7 Tropical & Subtropical Grasslands, Savannas & Shrublands  9 Flooded Grasslands & Savannas | Tropical grasslands |
| 4 Temperate Broadleaf & Mixed Forests  5 Temperate Conifer Forests | Temperate forests |
| 8 Temperate Grasslands, Savannas & Shrublands | Temperate grasslands |
| 6 Boreal Forests/Taiga | Boreal forests |
| 10 Montane Grasslands & Shrublands  11 Tundra | Alpine grasslands |
| 12 Mediterranean Forests, Woodlands & Scrub  13 Deserts & Xeric Shrublands | Dry biomes |

**Table S3** Factors affecting the natural logarithm of richness of different groupings of N-fixing prokaryotes, a comparison of the results from *nif*H and 16S SSU datasets. *NA* denotes the group could not be assessed with the denoted primer due to dataset limitations. Linear model (using Generalized Least Squares) parameter estimates with corresponding standard errors are shown, ² following the effect size denotes the effect of the quadratic term. P values are reported as follows: NS – not significant; * – p < 0.05; ** – p < 0.01; *** – p < 0.001.

|  | Total N-fixers | | Rhizobia | | Cyanobacteria | |
| --- | --- | --- | --- | --- | --- | --- |
|  | *nif*H | 16S SSU | *nif*H | 16S SSU | *nif*H | 16S SSU |
| Prokaryotic community  composition | 4.53±0.21  *** | *NA* | −0.42±0.19  * | −0.61±0.16  *** | 0.67±0.18  *** | 0.740±0.19  *** |
| Absolute latitude | 0.43±0.16  * | *NA* | 0.02±0.008  * | NS | NS | NS |
| pH | NS | *NA* | −4.93**²**±1.43  *** | −3.25**²**±1.15  ** | NS | −8.16±2.63  **  3.96**²**±1.81  ** |
| ln(P) | NS | *NA* | NS | NS | 4.52±1.64  ** | NS |
| ln(N) | −3.0**^2^**±1.12  ** | *NA* | NS | −3.40**²**±1.15  ** | −3.4^2^±1.27  ** | −7.61±1.44  *** |
| Bioclim PC1 [Stable hot] | 8.54±2.28  *** | *NA* | 18.32±2.67  *** | NS | −6.32±2.57  * | NS |
| Bioclim PC2 [Warm aridity] | NS | *NA* | −3.38**²**±1.38  * | NS | NS | NS |
| Bioclim PC3  [Seasonal humidity] | 4.34±1.18  *** | *NA* | 5.38±1.38  *** | 3.55±1.21  ** | NS | 3.40±1.41  * |
| Other soil macroelements [K, Ca, Mg] | NS | *NA* | NS | −4.19±2.09  * | NS | NS |
| N-fixing plant richness | NS | *NA* | NS | NS | NS | NS |
| Historical stability of biome | 0.49±0.21  * | *NA* | 0.50±0.24  * | NS | NS | NS |

**Table S4** Factors affecting the Shannon diversity index of different groupings of N-fixing prokaryotes, a comparison of the results from *nif*H and 16S SSU datasets. *NA* denotes the group could not be assessed with the denoted primer due to dataset limitations. Linear model (using Generalized Least Squares) parameter estimates with corresponding standard errors are shown, ² following the effect size denotes the effect of the quadratic term. P values are reported as follows: NS – not significant; * – p < 0.05; ** – p < 0.01; *** – p < 0.001.

|  | Total N-fixers | | Rhizobia | | Cyanobacteria | |
| --- | --- | --- | --- | --- | --- | --- |
|  | *nif*H | 16S SSU | *nif*H | 16S SSU | *nif*H | 16S SSU |
| Prokaryotic community  composition | NS | *NA* | −0.43±0.14  ** | −0.47±0.12  *** | 0.45±0.14  ** | 0.54±0.18  ** |
| Absolute latitude | 0.01±0.006  * | *NA* | 0.01±0.007  * | NS | −0.01±0.006  * | NS |
| pH | −3.01^2^±0.97  ** | *NA* | −4.44²±1.12  *** | 8.86±1.66  ***  −3.16²±0.91  *** | NS | −6.71±2.60  *  3.69**^2^**±1.41  ** |
| ln(P) | NS | *NA* | NS | NS | 3.97±1.32  ** | NS |
| ln(N) | −2.33^2^±0.88  ** | *NA* | −2.60²±1.01  * | 2.68±1.13  *  −3.49**^2^**±0.83  *** | −3.45±1.38  *  −2.76^2^±1.02  ** | −6.16±1.80  *** |
| Bioclim PC1 [Stable hot] | 7.28±1.82  *** | *NA* | 13.91±2.11  *** | 4.06±1.70  * | −5.02±2.07  * | NS |
| Bioclim PC2 [Warm aridity] | NS | *NA* | NS | NS | NS | NS |
| Bioclim PC3  [Seasonal humidity] | 3.98±0.95  *** | *NA* | 4.20±1.10  ***  2.08^2^±1.05  * | 2.42±0.88  ** | NS | 3.14±1.42  * |
| Other soil macroelements [K, Ca, Mg] | NS | *NA* | NS | NS | NS | NS |
| N-fixing plant richness | NS | *NA* | NS | NS | NS | NS |
| Historical stability of biomes | 0.54±0.17  ** | *NA* | 0.65±0.19  *** | 0.35±0.13  ** | NS | NS |

**Table S5** Factors affecting the community composition of different groupings of N-fixing prokaryotes, a comparison of the results from *nif*H and 16S SSU datasets. The community compositional analyses were made for the general prokaryotic 16S SSU dataset. *NA* denotes the group could not be assessed with the denoted primer due to dataset limitations. P values from Permutation tests for Distance-based Redundancy Analysis are reported as follows: NS – not significant; * – p < 0.05; ** – p < 0.01; *** – p < 0.001

|  | Total N-fixers | Rhizobia | | Cyanobacteria | |
| --- | --- | --- | --- | --- | --- |
|  | *nif*H | *nif*H | 16S SSU | *nif*H | 16S SSU |
| **dbRDA model adjusted R²** | **0.05** | **0.03** | **0.19** | **0.03** | **0.05** |
| Prokaryotic community  composition | *** | * | *** | NS | *** |
| Absolute latitude | *** | *** | NS | NS | ** |
| pH | *** | *** | *** | * | *** |
| ln(P) | NS | NS | * | NS | * |
| ln(N) | *** | ** | ** | NS | * |
| Bioclim PC1 [Stable hot] | *** | *** | *** | *** | *** |
| Bioclim PC2 [Warm aridity] | *** | * | ** | NS | ** |
| Bioclim PC3  [Seasonal humidity] | *** | *** | * | ** | NS |
| Other soil macroelements [K, Ca, Mg] | ** | * | NS | ** | NS |
| N-fixing plant richness | ** | ** | NS | NS | NS |
| Historical stability of biomes | NS | * | NS | NS | NS |
